# Structure and Functions of Endophytic Bacterial Communities Associated with *Sphagnum* Mosses and Their Drivers in Two Different Nutrient Types of Peatlands

**DOI:** 10.1007/s00248-024-02355-6

**Published:** 2024-02-26

**Authors:** Yue Wang, Dan Xue, Xuhui Chen, Qing Qiu, Huai Chen

**Affiliations:** 1 CAS Key Laboratory of Mountain Ecological Restoration and Bioresource Utilization & Ecological Restoration Biodiversity Conservation Key Laboratory of Sichuan Province, Chengdu Institute of Biology, Chinese Academy of Sciences, No. 9, Section 4, South Renmin Road, Chengdu, 610041 China; 2 Zoige Peatland and Global Change Research Station, Chinese Academy of Sciences, Hongyuan, 624400 China; 3 University of Chinese Academy of Sciences, Beijing, 100049 China

**Keywords:** Peat moss, Endophytic bacteria, Peatland, Methane oxidation, Nitrogen fixation, C/N cycling

## Abstract

*Sphagnum* mosses are keystone plant species in the peatland ecosystems that play a crucial role in the formation of peat, which shelters a broad diversity of endophytic bacteria with important ecological functions. In particular, methanotrophic and nitrogen-fixing endophytic bacteria benefit *Sphagnum* moss hosts by providing both carbon and nitrogen. However, the composition and abundance of endophytic bacteria from different species of *Sphagnum* moss in peatlands of different nutrient statuses and their drivers remain unclear. This study used 16S rRNA gene amplicon sequencing to examine endophytic bacterial communities in *Sphagnum* mosses and measured the activity of methanotrophic microbial by the ^13^C-CH_4_ oxidation rate. According to the results, the endophytic bacterial community structure varied among *Sphagnum* moss species and *Sphagnum capillifolium* had the highest endophytic bacterial alpha diversity. Moreover, chlorophyll, phenol oxidase, carbon contents, and water retention capacity strongly shaped the communities of endophytic bacteria. Finally, *Sphagnum palustre* in Hani (SP) had a higher methane oxidation rate than* S. palustre* in Taishanmiao. This result is associated with the higher average relative abundance of *Methyloferula* an obligate methanotroph in SP. In summary, this work highlights the effects of *Sphagnum* moss characteristics on the endophytic bacteriome. The endophytic bacteriome is important for *Sphagnum* moss productivity, as well as for carbon and nitrogen cycles in *Sphagnum* moss peatlands.

**Supplementary Information:**

The online version contains supplementary material available at 10.1007/s00248-024-02355-6.

## Introduction

Peatlands is a unique type of wetland ecosystem that occupies approximately 3% of the land area and stores about 600 Pg carbon, identifying it as an important carbon pool [1, 2]. However, global warming and changes of precipitation patterns have substantially undermined peatland stability [3–5]. For example, the speed of peat soil decomposition has accelerated [6] and emissions of methane (CH_4_) and carbon dioxide (CO_2_) have increased [7]. It is well known *Sphagnum* mosses are the “engineers” of peatlands; they largely increase long term carbon sequestration and greatly accelerate biogeochemical cycling. As a dominant bryophyte, *Sphagnum* mosses influences the surrounding environments as well as provides primary productivity to peatland ecosystems [8–12].

*Sphagnum* mosses are among the oldest nonvascular terrestrial plants and have adapted to highly acidic and nutrient deprived habitats that are often waterlogged and anoxic. By releasing H^+^ to acidify their surroundings or by generating phenolics and releasing them into the environment, *Sphagnum* mosses create an unfavorable environment for their competitors [11, 13]. These biochemical compounds can suppress the activity of extracellular enzymes of microorganisms and thus prevent the *Sphagnum* moss from being degraded [14], resulting in consistent accumulation of *Sphagnum* moss residues. Moreover, *Sphagnum* moss can activate aluminum and iron oxides in soils, thus facilitating the accumulation of mineral-associated organic carbon which is more stable in carbon poor environments [15]. *Sphagnum* mosses not only shape favorable microhabitats for their own growth and to maintain primary productivity [12], but also the *Sphagnum*-associated microbiome plays a critical role in the acquisition of nutrients and the protection against pathogenic bacteria [16, 17]. Water-filled hyaline cells are a special cell type accounting for ~90% of the volume of *Sphagnum* moss. Hyaline cells are essential for *Sphagnum* moss, as they provide the space for interactions between plant and the microbial community [18].

*Sphagnum* mosses possess a diverse microbiome including endophytic and epiphytic microorganisms [19, 20]. Especially, endophytes inhabiting hyaline cells are crucial to moss development and ecosystem function [21]. For example, *Sphagnum*-associated methanotrophic bacteria in hyaline cells can oxidize CH_4_ and provide additional carbon (C) to green cells engaged in photosynthesis [22–24]. In addition, N_2_-fixing prokaryotes fix nitrogen (N) from the atmosphere which is supplied to the plant host to compensate for the nitrogen deprived ecosystem [25–28]. Therefore, it is vital to better understand the relationship between *Sphagnum* mosses and microbiomes, especially endophytes that are not only involved in nutrient acquisition, but also those that impact C and N cycling in *Sphagnum*-dominated peatland ecosystems.

Several studies have shown that ecological factors are the main drivers that control the structure and function of the microbiome, such as the effectiveness of nutrients, pH, and temperature; these factors have a distinct association with the community composition of the microbiome [20, 29–31]. Moreover, a recent study reported that *Sphagnum* moss metabolites are an important predictor of the microbial community [32]. Physiological and biochemical properties of *Sphagnum* mosses can also shape the microbiome. However, many studies regarded both the endophytes and epiphytic microorganisms of *Sphagnum* mosses as a whole, and little is known about the specific role of endophytic bacteria. This study focused on the endophytic bacteria of different species of *Sphagnum* mosses, and clarified the microbiome community composition in detail. A previous study reported that plant species drive microbial communities [33], and the genotype of *Sphagnum* mosses may result in different structures and functions of endophytic bacteria. Moreover, endophytic bacteria are influenced by soil nutrient loading, temperature, and precipitation [34, 35]. Feather mosses are a dominant order in boreal forests and have an important nitrogen fixation capacity; however, moss-associated bacteria have been shown to vary between two dominant species, shaped by climatic, environmental, and nutritional factors [36]. However, it still remains whether endophytic microbial communities are affected by different types of peatlands, soil nutrients, or moss species, and which factors are crucial. To better understand the key factors affecting endophytic bacterial communities and the mechanism of how the endophytic bacteriome drives carbon cycling, an experiment was carried out involving two different typical peatlands. The dominant endophytic bacterial communities of *Sphagnum* mosses were analyzed, and the influences of *Sphagnum* moss physiological and biochemical factors as well as soil factors on these bacterial communities were assessed. Further, metagenomic metabolic functions and the C/N cycling the endophytic bacterial community is involved in were predicted.

A previous study suggested that the CH_4_ oxidizing ability varied between different species of *Sphagnum* mosses, although the inter-species influence was found to be small compared to that of habitats [37]. Furthermore, all *Sphagnum* moss species were shown to have the ability to support methanotrophic bacteria, but the methanotrophic activity was influenced by the water level [38]. However, these studies shared the limitation that sampling areas were located in the same climate and environmental zones. Therefore, one of the species in this study, *Sphagnum palustre*, was sampled in both ombrotrophic peatlands and poor minerotrophic fens. This experimental design was used to identify whether the same species of *Sphagnum* moss growing in different types of peatlands possesses the same CH_4_ oxidation activity, which was determined by activity assay. This approach better unlocks an understanding of how peatland types govern methane-oxidizing bacteria communities and their activity. The objectives of this study were to (1) examine the composition and structure *Sphagnum* moss endophytic bacteriome among different peatland types and species; (2) assess the influence of plant (*Sphagnum* moss physiological and biochemical) characteristics on these bacterial communities; (3) examine CH_4_ oxidation activity of the same species of *Sphagnum* moss between different peatland types (those sampled from Hani (SP) and those sampled from Taishanmiao (HSP)). In addition, PICRUST and FAPROTAX were used to predict the function of endophytic bacteria based on 16S rRNA genes.

**Fig. 1:**
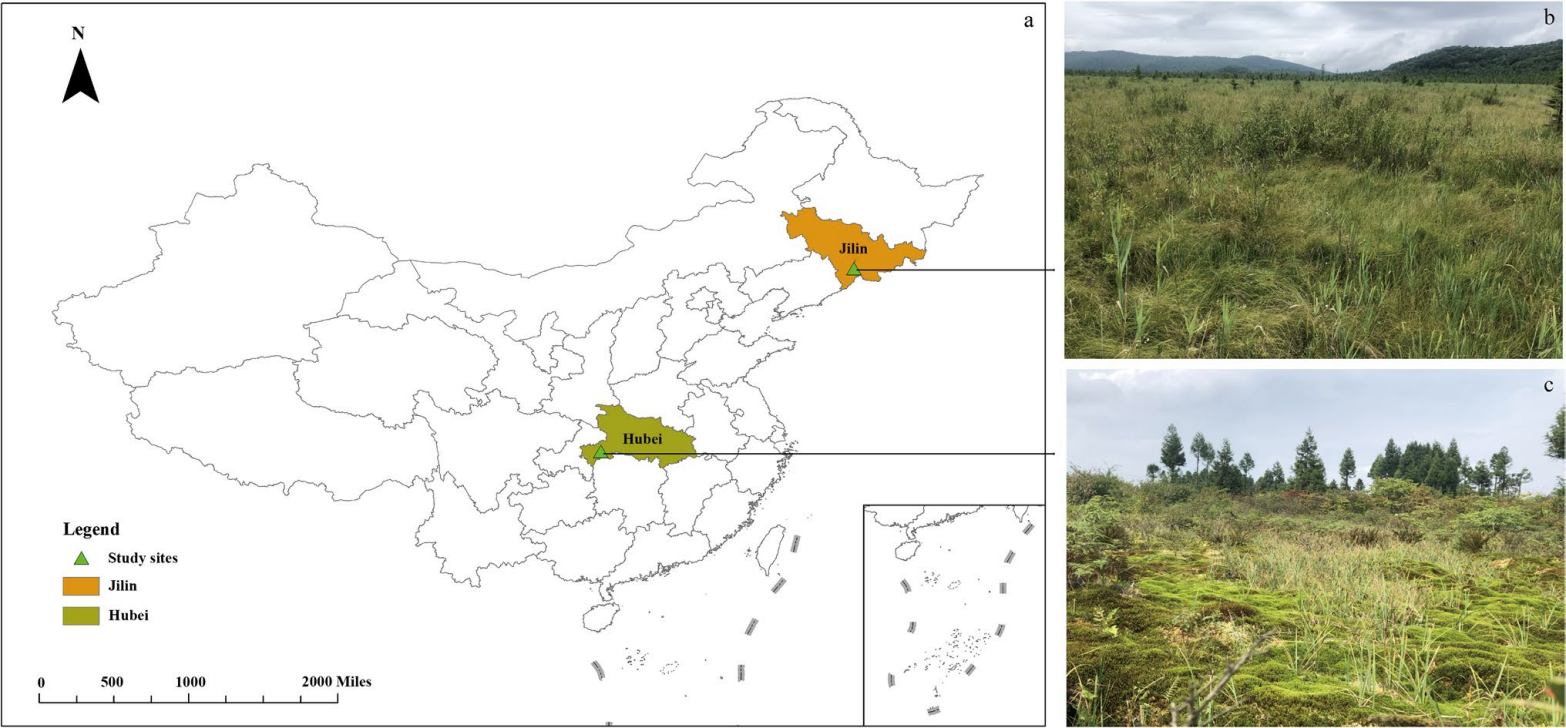
Sampling sites. Location of the study area in China (**a**). Sampling sites in the Hani peatlands (**b**) and Taishanmiao peatlands (**c**)

To reach these objectives, five *Sphagnum* moss species were collected: *S. palustre*, *S. magellanicum*, *S. fuscum*, and *S. capillifolium* were sampled from the Hani peatland, which is a temperate poor fen in the Changbai mountains, northeast China; *S. palustre* was sampled from Taishanmiao, which is a subtropical bog in southwest Hubei province, central China (Fig. 1). The endophytic microbial community was analyzed based on 16S rRNA marker genes, utilizing their metabolic and C/N element cycle functions. In addition, the incorporation of ^13^C-CH_4_ was measured to assess the activity of methanotrophic bacteria in *S. palustre*.

## Materials and Methods

### Study Sites

The *Sphagnum* moss samples were collected from two different types of peatlands (Fig. 1). Hani peatland is a poor minerotrophic peatland (i.e., a poor fen, where precipitation and underground water are the main sources of nutrients), located in the Changbai Mountains region of northeast China (42°13′05″N, 126°31′05″E, 900 m above sea level). Hani peatland is situated in the continental monsoon climate with a mean annual temperature of 3.9 °C and mean annual precipitation of 780 mm, respectively. The vegetation of Hani peatland includes shrubs, herbs, graminoids, and *Sphagnum* mosses. The dominant shrub is *Betula fruticose* Pall. var. *ruprechtiana* Trautv and herbs include *Eriophorum polystachion* L., *Carex lasiocarpa* Ehrh., and *Smilacina japonica* A. Gray [39]. Taishanmiao is an ombrotrophic peatland (i.e., a bog, where precipitation is the main source of nutrients) located in western Hubei province of central China (30°7′44″N, 109°47′12″E, 1800–1920 m above sea level), located in the subtropical subalpine region with a mean annual temperature of 7.20–8.27 °C, and a mean annual precipitation of 1768 mm. At Taishanmiao, vegetation types are mainly divided into shrubs, herbs, and mosses. *Enkianthus serrulatus*, *Rhododendron auriculatum*, *Carex taliensis*, and *Juncus effusus* are dominant shrub and herb species [40].

### Sample Collection and Processing

Because of the different development periods and environmental conditions, the dominant *Sphagnum* moss species in the two peatlands differed. *S. palustre*, *S. magellanicum*, *S. fuscum*, and *S. capillifolium* dominate Hani peatland [41], while Taishanmiao is dominated by *S. palustre* only. The habitats of these *Sphagnum mosses* are located in hummocks or midway between hummocks and hollows.

*Sphagnum* moss samples were collected into sterile zip lock bags and were transported to the laboratory with ice bag. *Sphagnum* moss samples were collected in triplicate. Upon arrival at laboratory, every sample was divided into three parts. In one part, the green parts were cut into pieces of 3–5 cm, the surface of which was sterilized for DNA extraction and sequencing; the other two parts were used to determine physicochemical indexes and stable isotope labeling activity assays. In addition, each kind of *Sphagnum* moss was incubated in a culture tray in an artificial climate chamber.

Peat soil samples (0–20 cm) were collected below the surface of growing *Sphagnum* mosses as the background value in two peatlands. The specific approach was to remove the vegetation cover and take peat cores by drilling; then, 0–20m peat soil cores were sliced and put into sterile bags. Peat soils samples were collected in three replicates in both Hani and Taishanmiao. Each sample was homogenized and stored at 4 °C until determination of physicochemical characteristics.

To extrac the DNA of *Sphagnum* moss endophytes, the samples were processed according to the following steps: First, the *Sphagnum* moss sample surface was slowly washed with tab water. Then, samples were placed into a beaker of 75% alcohol for 1 min and rinsed in sterile deionized water 5–6 times. Next, samples were immersed in 1% NaClO for 1 min and rinsed in sterile deionized water 7–8 times. Finally, using sterilized filter paper, the surface moisture of samples was dried. The final sterile water that had been used to rinse the samples was used to inoculate Rudolph culture medium. *Sphagnum* moss materials were frozen at −80 °C until DNA extraction [42, 43].

### Water-Holding Capacity

The water-holding capacity of *Sphagnum* mosses was characterized by measuring the water absorption until saturation. Three 2 cm × 2 cm quadrats of each species were placed into culture trays, and the green parts of the *Sphagnum* mosses capitulum were cut (approximately 3 cm). Samples were placed into a 50-ml centrifuge tube with 20 ml deionized water to adequately absorb water. Then, samples were taken out upside down until water dripping ceased, and the sample weight (W1) was recorded. Then, samples were placed in a drying oven at 72 °C and were dried to a constant weight 48 h and record dry weight (W2). Water-holding capacity = (W1 − W2)/W2 × 100%. The method has been described before [44] and adjustments were minor.

### Physiological and Biochemical Index

#### Carbon and Nitrogen Contents

*Sphagnum* moss samples (green segments of collected *Sphagnum* mosses) were dried in an oven at 105 °C for 30 min. Then, the temperature was adjusted to 72 °C, drying was continued for 48 h, and dried samples were ground with a ball mill. The dried and ground *Sphagnum* moss samples were used for subsequent analyses. C and N contents were determined using an elemental analyzer (CNS analyzer, EA 1110 Carlo Erba, Thermo Fisher Scientific, Waltham, MA, USA).

#### Chlorophyll Content, Total Phenols, Total Carbohydrates, Malondialdehyde, and Proline

Chlorophyll content was determined by the spectrophotometric method [45]. Briefly, fresh green capitulum of *Sphagnum* mosses (0.1 g) was put into a sterilized centrifuge tube with 80% (v/v) acetone solution (5 ml) to extract chlorophyll. The tube was wrapped with foil to protect it from light until the sample was extracted completely, followed by centrifugation for 10 min at 4 °C and 8000 rpm; then, the supernatant was absorbed. The supernatant (200 µl) was transferred into a 96-well plate to measure the absorbance value at 645 nm and 663 nm with a microplate reader (Thermo Scientific™ Varioskan™ LUX, Thermo Fisher Scientific, Waltham, MA, USA). Chlorophyll content was expressed in milligram of chlorophyll per a fresh weight (mg/g FW).

The total phenol content was determined by the Folin-Ciocalteu method [46]. First, dried *Sphagnum* moss sample (0.10 g) was extracted by 60% ethanol (2.5 ml) for 30 min and ultrasonicated for 30 min at 60 °C, followed by centrifugation for 10 min at 4 °C and 8000 rpm to obtain the supernatant. Then, Folin-Ciocalteu (250 µl) and 20% Na_2_CO_3_ (250 µl) were added to the supernatant (50 µl) of each sample. After incubation for 10 min, the absorbance was determined at 760 nm. Gallic acid was used as standard to calculate the total phenol content.

The total carbohydrates content was determined using the phenol-sulfuric acid method with minor modifications [47]. Fresh frozen *Sphagnum* moss samples (40 mg) were ground in liquid N; then, 50% methanol solution (4 ml) was added and the mixture was ultrasonicated for 40 min at 20 °C and 8000 rpm to obtain extracts. These extracts were shaken in an orbital shaker for 18 h at room temperature and 150 rpm, after which, the previous ultrasonication extraction step was repeated, followed by centrifugation for 5 min at 4 °C and 8000 rpm and absorption of the supernatant (final extract). Absorbed extracts (20 µl) were put into an Eppendorf tube to which distilled water (200 ml) was added for dilution, then 5% phenol solution (200 µl) and 98% H_2_SO_4_ (1 ml) were added. After incubation for 30 min and absorbance was determined at 490 nm. Glucose was prepared to draw the standard curve.

Malondialdehyde (MDA) is the product of lipid peroxidation in plants and the contents of MDA reflects the degree of lipid peroxidation. MDA was determined by the thiobarbituric acid method [48]. Fresh frozen *Sphagnum* moss samples (0.1 g) were ground in liquid N; then, 10% trichloroacetic acid (5 ml) was added, and the mixture was centrifugated for 10 min at 4 °C and 8000 rpm. Then, the supernatant (2 ml) was added to thiobarbituric acid (2 ml). After 15 min in the water bath, followed by centrifugation for 10 min at 4 °C and 8000 rpm, the absorbance was determined at 600, 532, and 450 nm.

Proline is a kind of osmoregulatory substance and the acid ninhydrin method was used to measure the proline content [49]. Briefly, the proline was extracted from dried *Sphagnum* moss samples (0.025 g) by 3% sulfosalicylic acid (1 ml), followed by centrifugation for 10 min at 4 °C and 4500 rpm to obtain the extracts. The supernatant (700 µl) was added to 5-ml glass tubes, and then the acid ninhydrin (2 ml) was added into the tube. In a water bath, samples were shaken gently to achieve uniform mixing. Absorbance was determined at 520 nm using l-proline as standard to calculate proline contents.

#### Enzyme Activity

The oxidative enzyme activities of phenoloxidase (PO O_2_) (PPO) and peroxidase (PO H_2_O_2_) (PER) were quantified [50, 51], using O_2_ and H_2_O_2_ as acceptors, respectively. First, enzymes were extracted: Green parts of fresh *Sphagnum* moss samples (3 g) were submerged in CaCl_2_ (50 ml, 0.10 M) with Tween 80 (0.05%) and polyvinylpolypyrrolidone (20 g) and the mixture was shaken for 1 h at room temperature. After centrifugation for 10 min at 4 °C and 10000 rpm, the supernatant was filtrated (1.2 µm, Waterman GF/D filters). Then, the enzyme activity was quantified. For PPO, concentrated extracts (150 µl) with 2,7-diaminofluorene (2 µl) in 96 wells microtiter plate. For PER, in addition to the above, 0.3% H_2_O_2_ (10 µl) was added to the rection system. PPO and PER were monitored at 600 nm by a microplate reader (Thermo Scientific™ Varioskan™ LUX, Thermo Fisher Scientific, Waltham, MA, USA). The enzyme activity was expressed as 1 nmol of substrate oxidized per min per mg of dry mass. In addition, another method (guaiacol method) was also used to determine peroxidase, expressed in POD [52]. Fresh sample (0.5 g) was added to 5 ml phosphate butter (pH = 5.6, containing 1% polyvinylpolypyrrolidone and 0.1% mercaptoethanol), followed by grinding in an ice bath. After centrifugation for 10 min at 4 °C and 10000 rpm, enzymes extracted with phosphate butter, 2% H_2_O_2_ and 2% guaiacol were determined by a spectrophotometer (FC-1100, Thermo Fisher Scientific, Waltham, MA, USA) at 470 nm. The absorbance was recorded every minute for five times in total. The enzyme activity was obtained by measuring the change in absorbance.

Glutamine synthetase activity was determined by the appropriate enzyme activity detection kit (Qiyi Biological Technology, Shanghai, China).

### Soil Physicochemical Analysis

After soil samples were processed including air-drying, grinding, and passing through a 2-mm sieve, total carbon (TC) and total nitrogen (TN) concentrations were determined by elemental analyzer (CNS analyzer, EA 1110 Carlo Erba, Thermo Fisher Scientific, Waltham, MA, USA). Before determining the soil total phosphorus (TP) of samples by elemental analyzer, the nitric acid and perchloric acid digestion method was used first to extract phosphorus from the sample to obtain an extraction solution. The dissolved organic carbon (DOC) concentration was determined by a total organic carbon analyzer (LIOYIL TOCII, Elementar, Germany). The peat soil water content was determined by gravimetric method. The pH value of the peat soil sample was measured by an acidity meter (OxyScan 300, UMS GmbH & Co. KG, Germany). Peat soil physiochemical properties are showed in Table S1.

### *CH*_*4*_* Oxidation Activity Assay*

To estimate the CH_4_ oxidation activity of *Sphagnum*-associated methane microbes in SP an HSP, the incorporation of the stable isotope (^13^C-CH_4_) in *Sphagnum* moss samples was measured [53]. Briefly, all test samples were in consistent vigorous growth condition (3-cm length of *Sphagnum* mosses capitulum). Into sterilized glass bottles (100 ml) with airtight plugs, three plantlets (of which the fresh weight was determined) were added and each species had three replicates. To all bottles, 5 ml (0.05%) ^13^C-CH_4_ was added to the headspace and controls were incubated without any labelled gassed. All samples were incubated in an artificial climate chamber for 48 h at 24 °C, including light/dark treatment (16 h with light and 8 h without light) and dark treatment (without light for 48 h), respectively. After this incubating step, samples were placed in oven at 72 °C for 48 h until a constant weight was reached (the dry weight) and ground by a ball mill (Retsch: MM 400). Then, the dried mass of each sample was weighted (approximately 4 mg) and put into tin cups. An elemental analyzer (CNS analyzer, Thermo Fisher Scientific) coupled to an isotopic ratio mass spectrometer (Finnigan Delta Plus, Thermo Fisher Scientific (Bremen) GmbH, Germany) was used to determine the fraction of ^13^C that had been incorporated into each sample. The activity of CH_4_ oxidation was expressed in nmol CH_4_ g^−1^ DW d^−1^.

### DNA Extraction, Amplification, and 16S rRNA Sequencing

Sterilized frozen samples were used for DNA extraction. The total genomic DNA of endophytic bacteria from *Sphagnum* moss samples was extracted using the OMEGA DNA Kit (M5635-02) (Omega Bio-Tek, Norcross, GA, USA) according to the manufacturer’s instructions. Using a NanoDrop NC200 spectrophotometer (Thermo Fisher Scientific, Waltham, MA, USA), the quantity and quality of samples were measured and 0.8% agarose gel electrophoresis was used to measure the integrity of the extracted DNA. The V5 and V7 regions of the microbial 16S rRNA gene were amplified using 799F (5′-AACMGGATTAGATACCCKG-3′)/1193R (5′-ACGTCATCCCCACCTTCC-3′) [54, 55]. PCR amplicons were purified with Vazyme Vahtstm DNA Clean Beads and quantified the by the Quant-iT PicoGreen dsDNA Assay Kit (Invitrogen, Carlsbad, CA, USA). Amplicons were pooled in equal amounts and pair-end 2250 bp sequences were used, obtained by Illumina NovaSeq platform with NovaSeq 6000 SP Reagent Kit (500 cycles) at Shanghai Personal Biotechbology Co., Ltd (Shanghai, China).

### Bioinformatics Analysis

The raw data were stored in FASTQ format and reads were performed with QIIME2 (2019.4) [56]. Raw paired-end reads primers and chimera were removed by the DADA2 plugin [57]. Quality filtering, denoising, and merging were also performed using DADA2. The sequence was processed by the above steps and dereplication. Using the cluster size module in Vsearch (v2.13.4_linux_x86_64) [58], the sequences with ≥ 97% similarity were clustered. The amplicon sequenced variants (ASVs) were aligned by MAFFT [59] and further used for annotation. Prokaryotic taxonomy was assigned with QIIME (2019.4), utilizing the Greengens database [60] (http://greengenes.secondgenome.com/) and Silva database [61] (http://www.arb-silva.de). ASV matrices were rarefied according to the sample with the least reads and depth was set to 95% of the lowest sequencing. All subsequent analyses were based on these rarefied data.

The Phylogenetic Investigation of Communities by Reconstruction of Unobserved States (PICRUSt2) [62] was used to predict the metabolic functions of marker gene sequence abundance in the samples based on the Kyoto Encyclopedia of Genes and Genomes (KEGG) database [63] (http://www.genome.jp/kegg/pathway.html). The database of Functional Annotation of Prokaryotic Taxa (FAPROTAX) [64] was also used to predict the function of prokaryote in *Sphagnum* mosses, with a specific focus on the processes of C and N elements cycling the members of the endophytic bacterial community are involved in. All original 16S rRNA sequences data were uploaded to the Sequence Read Archive of the National Center for Biotechnology Information (NCBI) database (accession number: PRJNA1006557).

### Statistical Analysis

Differences between *Sphagnum* moss endophytic microbiome (such as richness, Shannon’s diversity index, and Pileou’s evenness) were calculated in R with the *vegan* package. Non-metric multidimensional scaling (NMDS) with Bray-Curtis dissimilarity was used to identify the dissimilarities in endophytic bacterial communities. An analysis of similarities (ANOSIM) was conducted to examine the clustering of bacterial communities. Permutational multivariate analysis of variance model (PERMANOVA) was used to examine species-level differentiation in communities of endophytic bacteria. Canonical correspondence analysis (CCA) was performed to study the relationship between the relative abundance of the dominant endophytic bacterial communities as well as soil and plant variables (see Tables 1 and S1). Mantel test was used to examine the relationship between plant factors and the average relative abundance of the top 10 phylum, family, and genus endophytic bacteria communities. LEfSe analysis was used to identify biomarkers of endophytic microbial community in different *Sphagnum* mosses. The above analysis was carried out in R (4.1.3) using *phyloseq*, *DESeq2*, *ggcor*, *geosphere*, *tidyverse*, *microbiomeViz*, *ggtree*, *phyloseq*, and *vegan* packages, and figures were drawn using the *ggplot2* package.

**Table 1:** Physiological and biochemical characteristics of *Sphagnum* moss at Hani and Taishanmiao peatlands

Parameters	SP	SM	HSP	SC	SF
C (%)	41.25 ± 0.02c	40.33 ± 0.08e	43.10 ± 0.03a	40.63 ± 0.06d	41.94 ± 0.02b
N (%)	1.12 ± 0.01d	1.01 ± 0.01e	1.86 ± 0.01a	1.57 ± 0.01b	1.46 ± 0.01c
C:N	36.90 ± 0.20b	40.06 ± 0.20a	23.17 ± 0.18e	25.92 ± 0.16d	28.73 ± 0.18c
Chlorophyll (mg g^−1^)	1.87 ± 0.10b	1.67 ± 0.15b	1.20 ± 0.09b	2.65 ± 0.05ab	4.23 ± 0.91a
Total phenols (mg g^−1^)	0.63 ± 0.15a	0.46 ± 0.14a	0.72 ± 0.10a	0.52 ± 0.21a	0.44 ± 0.11a
Total carbohydrate (mg g^−1^)	66.91 ± 5.22c	91.59 ± 0.72ab	63.35 ± 12.95c	134.80 ± 18.57a	79.75 ± 5.23c
Proline (mg g^−1^)	0.81 ± 0.18a	0.96 ± 0.02a	1.02 ± 0.01a	1.01 ± 0.05a	0.83 ± 0.21a
MDA (nmol g^−1^)	1.04 ± 0.21a	1.04 ± 0.21a	1.45 ± 0.28a	1.60 ± 0.23a	1.34 ± 0.04a
Water retention capacity (%)	58.44 ± 5.98a	57.04 ± 9.18a	33.35 ± 3.90ab	57.68 ± 5.23a	29.44 ± 1.51b
GS (µmol·h^−1^·g^−1^)	2.91 ± 0.45ab	4.35 ± 1.16a	0.45 ± 0.05c	3.43 ± 0.46ab	4.70 ± 0.87a
PPO (mmol·min^−1^·mg^−1^)	6.62 ± 2.68a	1.30 ± 0.48a	2.80 ± 0.44a	4.32 ± 1.48a	2.80 ± 1.24a
PER (mmol·min^−1^·mg^−1^)	2.62 ± 0.54ab	2.58 ± 0.41ab	2.62 ± 0.54ab	1.81 ± 0.38b	3.72 ± 0.27a
POD (mmol·min^−1^·mg^−1^)	0.69 ± 0.08b	4.11 ± 0.46a	2.05 ± 0.42ab	0.33 ± 0.17b	3.38 ± 0.77a

Values are mean ± SE (*n* = 3). Values with the lowercase letters in the same row indicate significant differences at *p* < 0.05 level. SM, SC, SF, and SP represent *Sphagnum magellanicum*, *Sphagnum fuscum*, *Sphagnum capillifolium*, and *Sphagnum palustre* which was sampled in Hani; HSP represents *Sphagnum palustre* which was sampled in Taishanmiao. *C,* carbon contents; *N,* nitrogen contents; *,* *PPO,* phenol oxidase (PO O_2_); *PER,* peroxidase (PO H_2_O_2_); *POD,* peroxidase; *GS,* glutamine synthetase

Spearman’s correlation test was calculated by SPSS 18.0. Soil and plant parameters were normality tested before analysis. Differences in physiological characteristics between different species of *Sphagnum* mosses were evaluated using one-way ANOVA and multiple comparisons in SPSS 18.0. The results of predicting prokaryote C/N element cycling and metabolic pathway function were tested with the Kruskal-Wallis test.

## Results

### Physiological and Biochemical Characteristics of Sphagnum Mosses

The physiological and biochemical characteristics of *Sphagnum* mosses showed significant differences among *Sphagnum* mosses, especially the contents of C, N and total carbohydrate (Table 1). C and N in *Sphagnum palustre* sampled from Taishanmiao (HSP) were 43.10% and 1.86%, respectively, and were the highest among these samples (*P* < 0.05, Table 1); while C and N in *Sphanum palustre* sampled from Hani (SP) were 23% and 30%, respectively (Table 1). The chlorophyll content of *Sphagnum fuscum* (SF) was 4.23 mg/g, which was significantly higher compared to other species of *Sphagnum* mosses (*P* < 0.05, Table 1). *Sphagnum capillifolium* (SC) had the highest concentration of total carbohydrates (134.80 mg/g) while HSP had the lowest concentration (63.35 mg/g) (Table 1).

*Sphagnum* species from Hani had similar water-holding capacities except for SF (29.44%) (*P* < 0.05, Table 1) and SP (58.44%) which had higher capacities than HSP (33.35%). These results showed that the contents of proline and MDA did not vary significantly between species. The activity of POD had clear differences among different moss species, where the highest was SM with 4.11 mmol·min^−1^·mg^−1^, followed by SF (3.38 mmol·min^−1^·mg^−1^), and SC was the lowest (0.33 mmol·min^−1^·mg^−1^) (Table 1).

### Endophytic Bacterial Community Structure and Composition in Different Sphagnum Species

In total, 33,481 ASVs were obtained in all samples after quality control and rarefaction. The rarefaction curves across all samples almost reached stable values (Fig. S1), indicating that most of the endophytic bacteria in *Sphagnum* moss samples were captured.

**Fig. 2:**
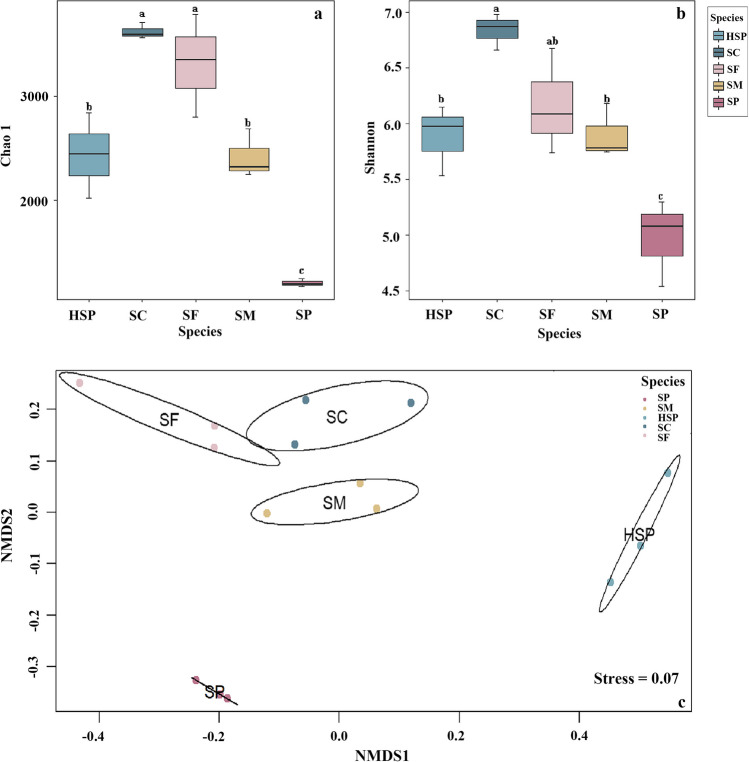
Endophytic bacterial communities in *Sphagnum* moss. Alpha diversity of 16S rRNA gene in different species *Sphagnum* mosses including Chao1 index (**a**) and Shannon index (**b**). Non metric multidimensional scaling (NMDS) ordinations of prokaryotic ASV compositions among different *Sphagnum* moss species (**c**). Different color points represent different species of *Sphagnum* mosses. Significant differences (*p* < 0.05) are indicated with lowercase letters. SM, *Sphagnum magellanicum*; SF, *Sphagnum fuscum*; SC, *Sphagnum capillifolium*; HSP, *Sphagnum palustre* (sampled from Taishanmiao); SP, *Sphagnum palustre* (sampled from Hani)

Based on ASVs, alpha diversity indexes of endophytic bacteria were estimated using Shannon, Chao1 (Fig. 2a, b) and Pielous evenness indexes (Table S2). Alpha diversity was the highest in SC, followed by SF and SP had the lowest alpha diversity (*P* < 0.05, Fig. 2 and Table S2). Between HSP and SP, Chao1 and Shannon diversity indexes were significantly different (*P* < 0.05, Fig. 2a, b).

NMDS analysis showed the dissimilarities in endophytic bacterial communities based on the Bray-Curtis matrix. Different *Sphagnum* moss species were distinguished by differences in their prokaryotic ASV composition (Fig. 2c), and communities of the same *Sphagnum* moss species clustered together. Notably, the endophytic communities of HSP were separated from the other four groups on the first axis, and the endophytic communities of SP were completely separated from the other three species from Hani on the second axis (Fig. 2c). ANOSIM test showed that the endophytic communities differed significantly between *Sphagnum* moss species (*R* = 0.9481, *P* = 0.001). PERMANOVA was further used to examine species-level differentiation in communities of endophytic bacteria (*F* = 3.832, *P* < 0.001).

**Fig. 3:**
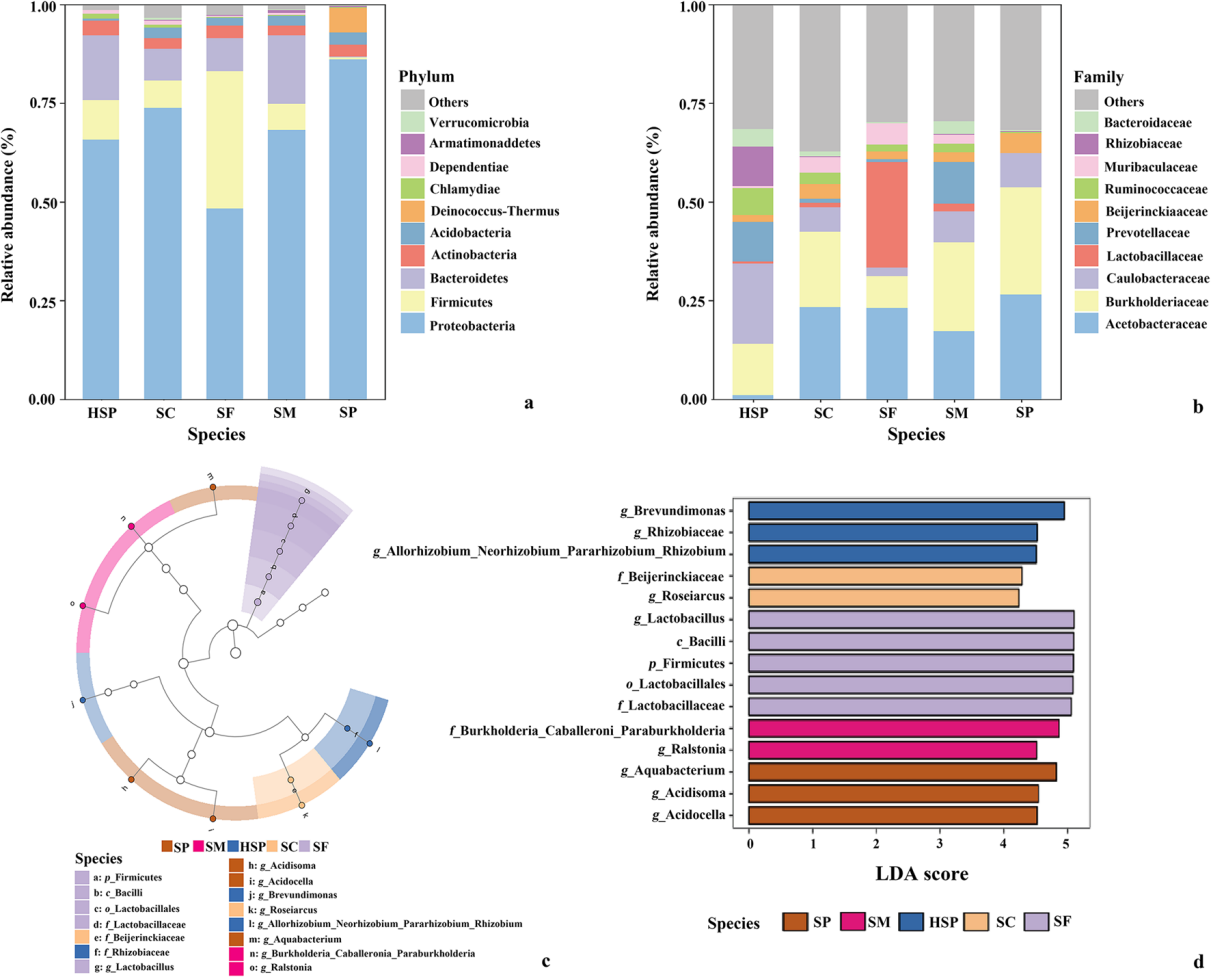
*Sphagnum* moss endophytic bacterial community composition. Average relative abundance of the top 10 phylum (**a**) and family (**b**) in different species *Sphagnum* mosses. Taxonomic cladogram (**c**) through linear discriminant analyzes effect size (LEfSe) and biomarker of endophytic bacterial communities in different species *Sphagnum* mosses with LDA SCORE > 3 (**d**). Significant discriminant taxa of SP (*Sphagnum palustre* was sampled from Hani), SM (*Sphagnum magellanicum*), SF (*Sphagnum fuscum*), SC (*Sphagnum capillifolium*), and HSP (*Sphagnum palustre* was sampled from Taishanmiao) are colored in brown, pink, purple, orange, and blue, separately. Colorless nodes represent taxa that do not significantly discriminate among *Sphagnum* mosses. The dimension of nodes is positively correlated with the relative abundance of the taxon

To clarify the composition of the endophytic bacteria community among the sampled *Sphagnum* mosses, the relative abundances of the top 10 bacterial phyla, families, and genera were analyzed. In total, 36 phyla were identified from all sequences and the most abundant top 10 phyla are depicted in Fig. 3a. The endophytic bacterial community in *Sphagnum* mosses was mainly composed of Proreobacteria, Actinobacteria, Acidobacteria, Firmictes, and Bacteroidetes; however, the proportions differed (Fig. 3a and Table S3). Proteobacteria was more abundant in SP than in other mosses, accounting for 86.00% of the total relative abundance, while Bacteroidetes and Firmicutes were the lowest among all moss samples (Fig. 3a and Table S3). The average relative abundance of Acidobacteria in HSP was 0.40% lower than in other species, while Chlamydiae were significantly more abundant in HSP compared to other species (*P* < 0.05, Table S3). At the family level, the average relative abundance of Acetobacteraceae was higher in SP (*P* < 0.05, Fig. 3b and Table S3). Conversely, Rhizobiaceae were significantly higher in HSP (10.05%) than in other *Sphagnum* mosses (*P* < 0.05, Fig. 3b and Table S3). The average relative abundance of Beijerinckiaceae was 5.10% in SP, which is significantly higher compared to HSP (*P* < 0.05, Fig. 3b and Table S3). Moreover, the endophytic microbiome between SP and HSP had distinct genera (Fig. S2); an example can be found in the average relative abundances of the *Acidocella* and *Bacteroides* (*P* < 0.05, Table S3).

To identify biomarkers of the endophytic microbial community in different *Sphagnum* moss, LEfSe analysis was performed (Fig. 3c, d). According to the results of LDA score (LDA > 3, *P* < 0.01, Fig. 3d), SF had five discriminative biomarkers from phylum to genus, which were affiliated with phylum Firmicutes, class Bacilli, order Lactobacillales, family Lactobacillaceae, and genus *Lactobacillus*. In SC samples, two biomarkers were family Beijerinckiaceae and genus *Roseiarcus*. HSP and SP had different biomarkers; according to the evolutionary clustering analyses diagram, Rhizobiaceae were abundant in the blue parts, representing HSP, while Aquabacterium were most abundant in the brown parts, representing SP (Fig. 3c).

### Relationship Between Phytochemical Parameters and Endophytic Bacterial Abundances in Sphagnum Mosses

**Fig. 4:**
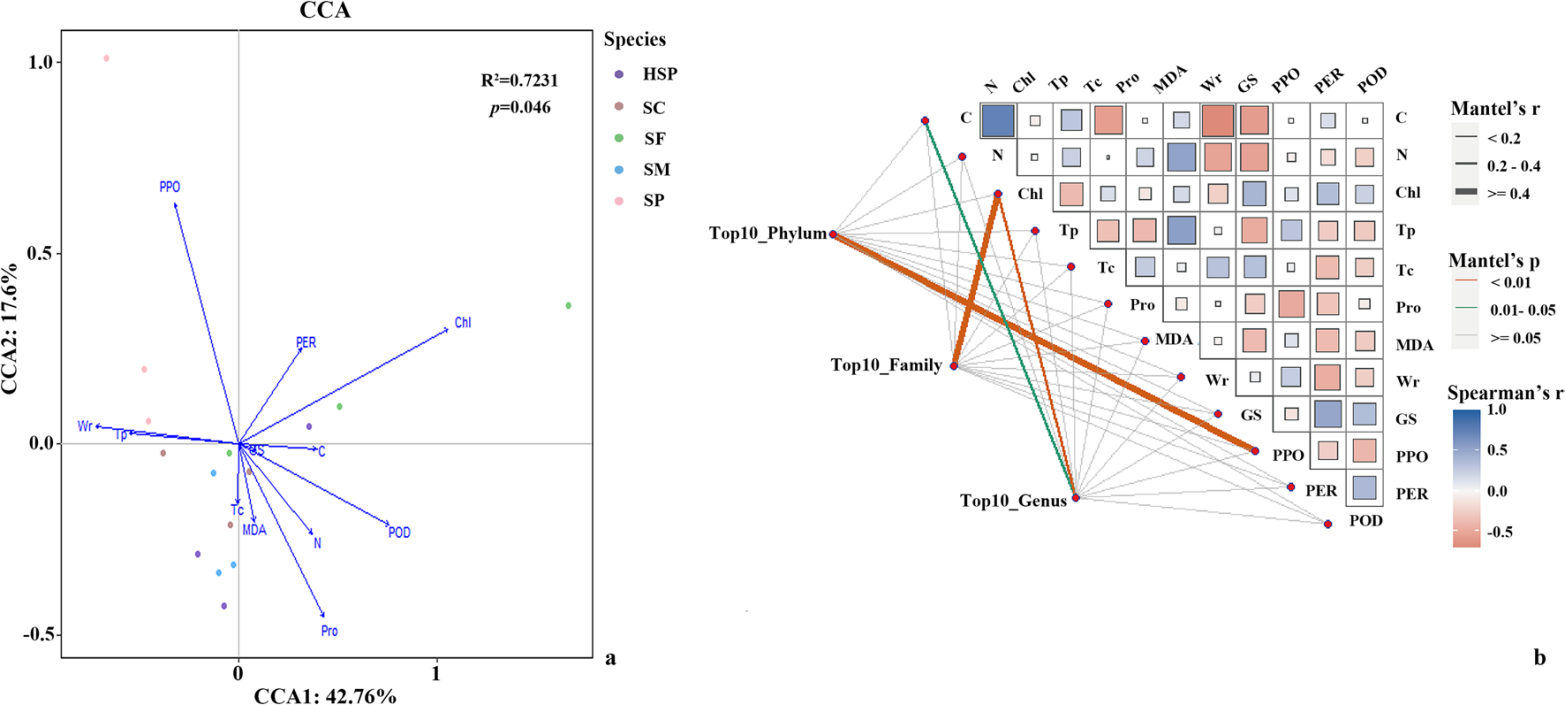
Correlation between plant phytochemical parameters and bacterial communities. Canonical correspondence analysis of the relative abundance of the top 10 phyla endophytic bacteria in association *Sphagnum* moss characteristics (**a**) and correlation between *Sphagnum* moss physiological and biochemical characteristics and the relative abundance of the top 10 phyla, families, and genera of endophytic bacteria (**b**). C, carbon contents; N, nitrogen contents; Chl, chlorophyll contents; Tp, total phenol contents; Tc, total carbohydrates; Pro, proline; MDA, malondialdehyde; Wr, water retention capacity; PPO, phenol oxidase (PO O_2_); PER, peroxidase (PO H_2_O_2_); POD, peroxidase; GS, glutamine synthetase

To better understand the linkages between plant physiological and biochemical characteristics and endophytic bacteria communities, CCA and Mantel test were used to detect important environmental factors that influence the relative abundance of the top 10 phylum, family, and genera of endophytic bacteria. CCA analysis showed that the correlation between chlorophyll contents, water-holding capacity, and PPO activity and endophytic bacteria communities at the phylum level (*R*^2^ = 0.7231, Fig. 4a). Similarly, chlorophyll contents and water-holding capacity also shaped the relative abundance of the top 10 family and genera endophytic bacteria in *Sphagnum* mosses (Fig. S3, Table S5and Table S6). Significant Spearman’s correlations were found between the relative abundance of Proteobacteria and water-holding capacity, while Firmicutes were negatively correlated with water retention capacity (*P* < 0.05, Table S4). Chlorophyll contents were identified as a crucial factor affecting the endophytic bacterial communities at the family level. The relative abundance of Acetobacteraceae and Muribaculaceae were significantly positively correlated with chlorophyll contents (*P* < 0.05, Table S5).

Moreover, the Mantel test also presented correlations between plant parameters and the microbial community (Fig. 4b). Significant correlation were found between the relative abundance of top the 10 genera endophytic bacteria and C contents (Mantel’s *r* = 0.303, *P* = 0.017), and the relationships with chlorophyll content (Mantel’s *r* = 0.459, *P* = 0.006); further, the chlorophyll content correlated significantly with the top 10 families of endophytic bacteria communities (Mantel’s *r* = 0.492, *P* = 0.003), and correlated significantly with the relative abundance of the top 10 phylum endophytic bacteria and PPO (Mantel’s *r* = 0.448, *P* = 0.01).

### Endophytic Bacterial Communities Enrich Functional Roles Related to C Cycling

**Fig. 5:**
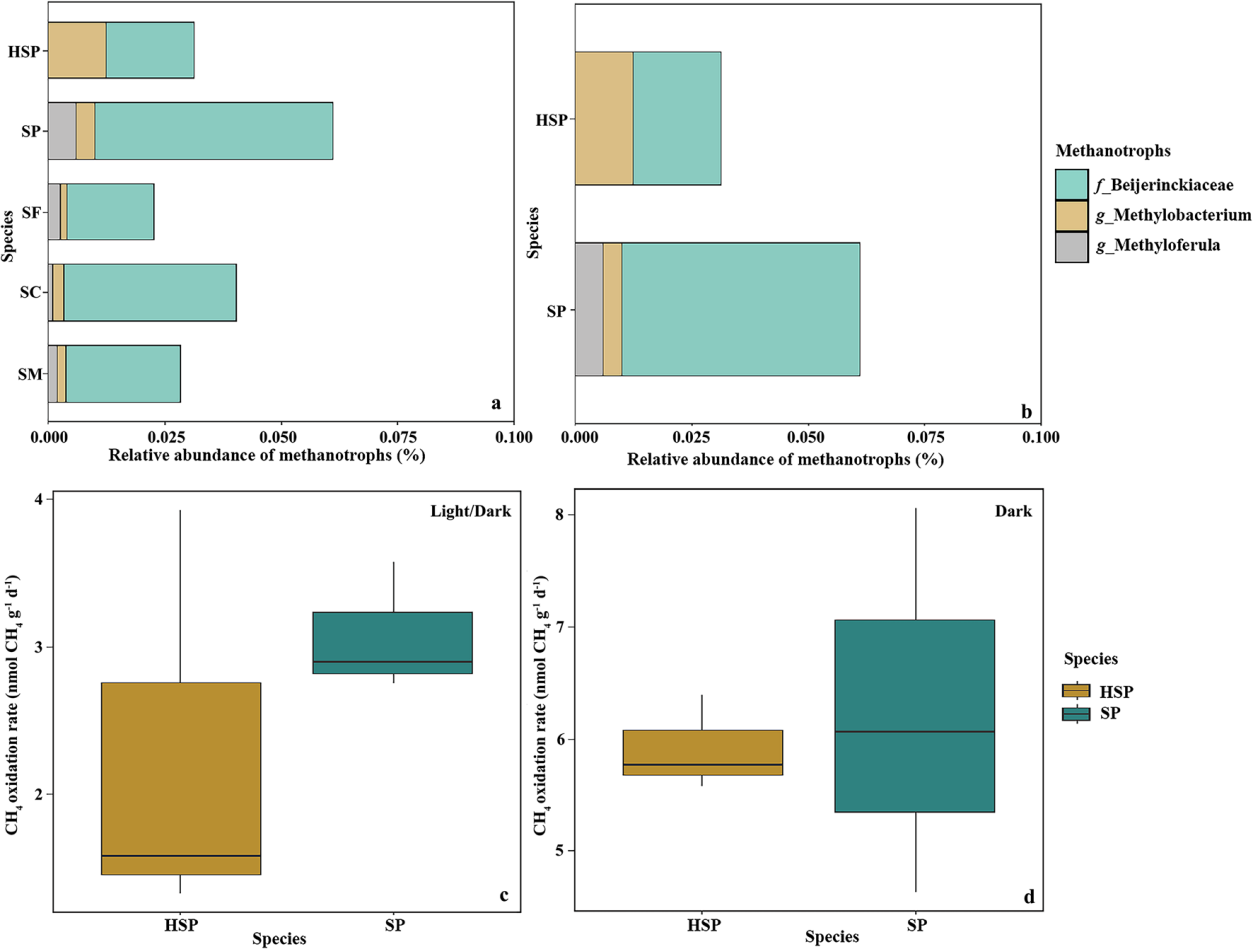
Comparison of the relative abundances of methanotrophic prokaryotic taxa functions. The relative abundances of methanotrophic in five *Sphagnum* mosses (**a**), *Sphagnum palustre* was sampled from Hani (SP) and Taishanmiao (HSP), respectively (**b**). Methane oxidation activity (represente by ^13^C-CH_4_ incorporation rate) of *Sphagnum palustre* was sampled from Hani (SP) and Taishanmiao (HSP) at light/dark (**c**) and dark treatments (**d**), respectively. Light/dark treatment represents samples kept in artificial climate chamber for 16 h with light and 8 h without light at 24 °C. Dark treatment represents samples kept in artificial climate chamber without light for 48 h at 24°C

Based on 16S rRNA gene taxonomic analysis, the functions of putative methanotrophic prokaryotic taxa are discussed. Methanotrophic communities at the family or genus level varied between different *Sphagnum* moss species (Fig. 5a and Table S7). The relative abundance of Beijerinckiaceae family comprised 5.10% of the total endophytic bacteria communities in SP; at the genus level, SP had the highest relative abundance of *Methyloferula* (family Beijerinckiaceae). The relative abundance of *Methyloferula* in *Sphagnum* mosses from Hani was higher than in mosses from Taishanmiao. However, the genus of *Methylobacteriu* accounted for 1.24% in HSP, which was the highest among all *Sphagnum* mosses. Moreover, methanotrophic communities showed differences between HSP and SP, as depicted in Fig. 5b.

Because the methanotrophic communities show differences between SP and HSP (see Fig. 5b and Table S9), whether these differences influence the methane oxidation efficiency was examined. An activity experiment was conducted to explore whether there were differences between SP and HSP (Fig. 5c, d). The CH_4_ oxidation rate of methanotrophic communities in SP (3.0702 ± 0.2554 nmol CH_4_ g^−1^ DW d^−1^) was higher than that of HSP (2.2752 ± 0.8289 nmol CH_4_ g^−1^ DW d^−1^) (Fig. 5c and Table S9). Similarly, SP was also higher than HSP in the dark treatment (Fig. 5d and Table S9). However, no significant difference was found between HSP and SP.

### Element Cycling (C and N) and Prediction of Metabolic Pathway Function

**Fig. 6:**
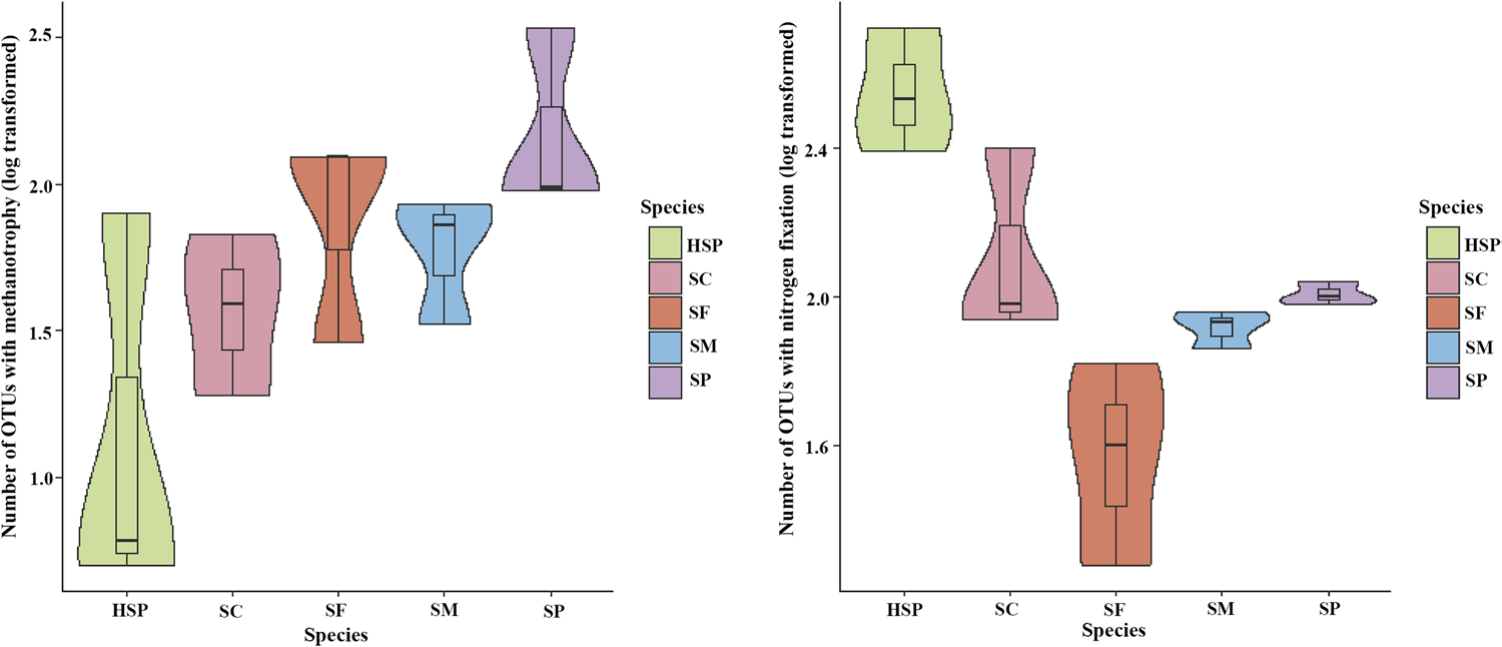
The number of ASVs of methanotrophs function (**a**) and nitrogen fixation (**b**) in *Sphagnum* moss based on FAPROTAX prediction

**Fig. 7:**
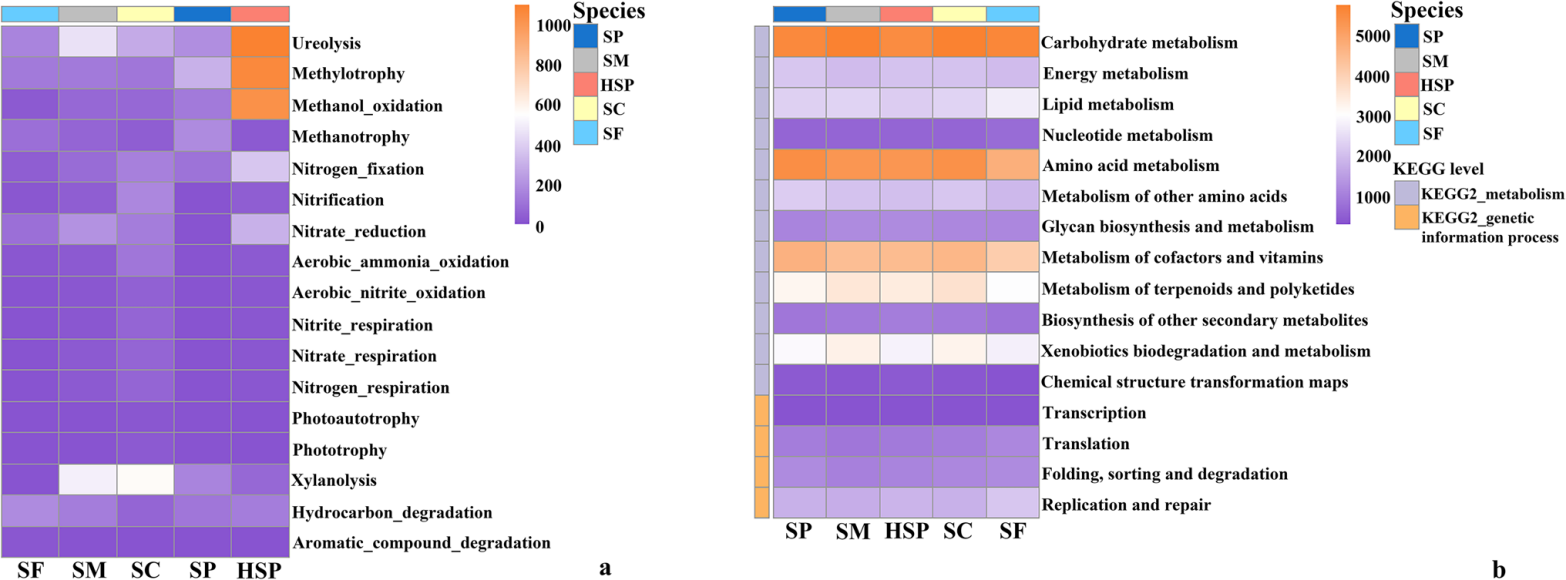
Element cycling functional genes and potential metabolic pathways prediction. Heatmap of C and N cycling functions of endophytic bacteria based on FAPROTAX prediction (**a**). Heatmap of metabolism and genetic information process pathways (KEGG II) of endophytic bacterium between *Sphagnum* mosses by PICRUSt2 (**b**). Data were based on number of ASVs of each function in *sphagnum* moss

Given the key role endophytic microbes play in *Sphagnum* mosses, combined with functions and activity assays of putative methanotrophic prokaryotic taxa, functional annotation of prokaryotic taxa was performed (FAPROTAX) to analyze the putative function of the *Sphagnum* microbiome (Fig. 7a). Most of the functional genes showed similar abundances across different *Sphagnum* species. The average functional abundances of methylotrophy, methanol oxidation, and ureolysis were higher in HSP than in other mosses. The average functional abundance methanotrophy in SP was the highest among all *Sphagnum* moss samples, followed by SF. Specifically, the number of ASVs of methanotrophy in SP was approximately twice that of HSP (Fig. 6a).

In addition, microbial functions were also involved in N cycling (e.g., nitrogen fixation, nitrification, and nitrate reduction). Nitrogen fixation differed significantly among sampled mosses; the average functional abundance of nitrogen fixation was higher in HSP than in other species (Fig. 7a), and the number of ASVs involved in nitrogen fixation was also higher in HSP (Fig. 6b). Combined with putative diazotrophic communities based on 16S rRNA gene taxonomic analysis (Fig. S4), diazotrophic communities included Magnetospirillaceae, Sphingomonadaceae, Rhizobiaceae, Pseudomonadaceae, and Beijerinckiaceae. The relative abundance of Rhizobiaceae was the highest in HSP among all sampled mosses, and the relative abundance of Sphingomonadaceae within all endophytic bacterium communities was 3.11 ± 0.01% in SP (Table S5). Furthermore, the relative abundance of Beijerinckiaceae (including C and N cycling functional microorganisms) was also the highest in SP. Correlation analysis was conducted between the function predicted by FAPROTAX and the relative abundance of the top 10 family endophytic bacteria (Table S10). These results showed that Rhizobiaceae exhibited a highly positive Spearman’s correlation with the functions of N fixation (*P* < 0.01), ureolysis (*P* < 0.01), and methanol oxidation (*P* < 0.01, Table S10).

PICRUSt2 was used to predict the potential metabolic functions of the endophytic microbial community. The results showed that different species of *Sphagnum* mosses had similar relative abundances on the KEGG level I pathway (Fig. S5). Metabolism and genetic information process pathways differed significantly between *Sphagnum* mosses (*P* < 0.05) (Table S11). Metabolic pathways including 12 pathways in level II, the relative abundance of carbohydrate metabolism, amino acid metabolism, as well as the cofactors and vitamins metabolism were elevated in every species moss (Fig. 7b). Metabolism of terpenoids and polyketides differed significantly between species (*P* < 0.05, Kruskal-Wallis test, Table S11,) and SC was the highest.

## Discussion

Endophytic bacteria have great implications to the growth and development of *Sphagnum* mosses, and the mosses, in turn, influence the C and N cycles in the peatland ecosystem [11, 65]. This study demonstrated the differences of endophytic bacteria communities in *Sphagnum* mosses across two sites and various species. These differences have important implications for clarifying the endophytic microbial community structure, thus contributing to a better understanding of the function of endophytic microbes. This knowledge furthers research on how to promote *Sphagnum* moss growth and its development under climate change, as well as on how to evaluate C and N cycling functions in peatlands. Previous studies reported that plant-associated microbiomes are influenced by both biotic and abiotic factors [25, 66, 67]. Similarly, endophytic bacteria are also influenced by many factors including environmental conditions, plant species or genotypes, and even growth periods [68, 69].

In this study, differences were found among the sampled *Sphagnum* moss species regarding physiological and biochemical characteristics. *S*. *palustre* (HSP and SP) grown in different types of peatlands also showed physiological differences (Table 1). *Sphagnum* moss species also differed in morphological traits, for example, the shape and size of leaves in the capitate branch, which is a critical water retention characteristic for mosses [18]. Microbiomes are shaped by plant host species, microbial interactions, and environmental factors including latitude, precipitation, and soil nutrients [70–72]. These factors also affect the endophytic microbiome to a certain extent. Indeed, the characteristics of the sampled *Sphagnum* moss species significantly affected the structure of endophytic bacterial communities (Fig. 4). *Sphagnum* moss gametophytes can acquire specific microbiomes from sporophytes which feature abundant bacterial diversity [19], emphasizing that host specificity and genetic factors are determinants of the moss plant microbiome. The identity of host species influences bacterial gene expression, and it was proposed [73] that feather moss can upregulate certain genes, thus promoting cyanobacterial abundance and growth to a certain degree. Except for species identity, in this study, remarkable correlation between the relative abundance of the top 10 phyla or families of endophytic bacterial communities in *Sphagnum* moss and water-holding capacity, chlorophyll contents, and the activity of PPO (Fig. 4). These factors vary with *Sphagnum* moss species, and can further influence endophytic bacterial communities. Chlorophyll, water, and CO_2_ are indispensable for photosynthesis. On the one hand, abundant bacteria associated with methane oxidation in both the green photosynthetic cells and hyaline cells of *Sphagnum* mosses provide additional CO_2_ thus guaranteeing supplementation with sufficient CO_2_ [24, 74]. On the other hand, sufficient water showed that the *Sphagnum* moss hyaline cells perform well. Porous hyaline cells not only play a vital function as water storage organs and transport water to adjacent green photosynthetic cell but also provide a habitat for an abundance of microbial communities [18, 19]. Hence, this also reflects the positive correlation between the dominant microbiome at the phylum and family levels with chlorophyll contents. Water-holding capacity was another important factor for shifts in endophytic bacterial communities. The results of Spearman correlation test indicated that there is a positive relationship between water-holding capacity and the family of Burkholderiaceae (Spearman’s *ρ* = 0.564, *P* < 0.05). The members of Burkholderiaceae are distributed throughout a variety of habitats including animals, plants, and soil [75], and this community adapted acidic peatlands [76]. Previous studies have shown that in *Sphagnum* moss, both endophytic and entophytic bacteria, possess a high diversity of the genus of *Burkholderia*. *Burkholderia* was dominant in *S. magellanicum* and *S. fuscum* [17], and ingredients with antifungal activities were detected in *Burkholderia* species [77].

Global warming will change the stability and carbon sequestration may suffer from a series of influences. These influences can be partly attributed to the change of the activity of extracellular phenol oxidases which could degrade complex polyphenols and play important roles in the soil carbon cycle in the peatland ecosystem [78]. In this study, the PPO activity did not show differences between the sampled *Sphagnum* moss species, while the activities of PER between SC and SF were significantly different (Table 1). Previous research examined the activities of PPO and PER in *S. fuscum* in *Sphagnum*-dominated peatlands under simulated warming climate; the findings showed that warming treatment also did not alter PPO activity but increased PER activities in the living top segments [79]. Overall, the differences in the phenol oxidases activity between different species of *Sphagnum* mosses may be related to differences of endophytic bacteria. Activity is also affected by abiotic factors, and phenol oxidase activity was positively related to the moisture content [80].

Among these *Sphagnum* moss species, HSP and SP belong to the same species but grow in different peatland types and show differences in bacterial composition at the phylum level. For example, the relative abundances of Bacteroidetes (HSP, 16.46%; SP, 0.43%) and Firmicutes (HSP, 9.97%; SP, 0.40%) (Fig. 3a and Supplemental Table S3). The LEfSe results also showed that Rhizobiaceae were abundant in HSP while Aquabacterium were the most abundant in SP (Fig. 3c, d). Prior studies reporting that as long as the same species of moss was sampled in the same ecological amplitude, the moss-associated microbial composition was similar [19, 26]. Moreover, in comparison to other species sampled in Hani (i.e., SC, SF, and SM), SP still showed differences. According to a previous observation, *S. fuscum* (hummock) and *S. angustifolium* (broader ecology), which grow in the same bog ecosystem, show a high degree of similarity [17, 20]. These discrepancies can partially be explained as follows: in each of the experiments, the research object differ. More than just endophytes in sampled *Sphagnum* mosses bacteriome in their study, the diversity of the microbial community on plant surface is generally higher than the diversity of the endophytic bacteria [81].

In this study, active methanotrophic endophytic bacteria in *Sphagnum* mosses include *Methyloferula* and *Methylobacterium*. A previous study identified *Methylferula* as one of the most active methanotrophs associated with *Sphagnum* mosses in peatland [82]. The relative abundance of *Methyloferula* in SP was higher than that of other mosses and the activity assay results further showed that the CH_4_ oxidation rate was higher in SP than in HSP (Fig. 5c, d). This result suggests that *Methyloferula* has mainly methanotrophs function in the *Sphagnum* moss endophytic bacteriome. Beyond *Methyloferula*, the relative abundance of Beijerinckiaceae was higher in SP than in other species including HSP (Table S7). Beijerinckiaceae comprise obligate methanotrophs, facultative methanotrophs, and facultative methylotrophs. Moreover, in line with previous studies, the higher abundance and diversity diazotrophs could contribute to higher N_2_-fixation rates, emphasizing that the higher microbiome diversity will promote the microbiome function [36, 83, 84]. At the same time, methanotrophs have been shown to provide C derived from atmospheric CH_4_ to *Sphagnum* mosses [38], but the rate of oxidation varied among *Sphagnum* moss species. However, it has been suggested that CH_4_-oxidizing bacteria associated with *Sphagnum* mosses are not influenced by the *Sphagnum* moss species [85], but rather, are controlled by abiotic factors [38, 86]. In the present study, both HSP and SP were collected from a moderately rich fen in northeast China and a poor nutrient bog located in a subtropical alpine region (Fig. 1), respectively; the results highlight the differences between SP and HSP (Fig. 5c, d). In addition, the rate of CH_4_ oxidation was higher in the dark than in the light/dark treatment, which differed from previously published results showing that light can stimulate of CH_4_ oxidation activity [87]. Moreover, physiologic characteristics of *Sphagnum* moss can influence the number of endophytic CH_4_-oxidizing bacteria in plant tissue and thus the CH_4_ oxidation capacity [37]; the reason is that two different species of *Sphagnum* mosses with physiologic characteristics differences growing at the same site showed different CH_4_ consumption levels. Microbial communities growing in similar environments were found to have similar functions [88] in *Sphagnum* mosses; despite, these differences in microbiome, functional redundancy in CH_4_ oxidation is a widespread mechanism in peatland, the higher rate in SP may be the result of the higher diversity and activity of functional endophytic microbes in cells.

Based on the relative abundances results of nitrogen fixation prokaryotic taxa in the endophytic bacteriome of *Sphagnum* mosses, it can be inferred that the N fixing efficiency differed among the sampled mosses. It has been shown that the order of Rhizobiales contains nitrogen-fixing or methane-oxidation microbiomes [89, 90] that play a crucial role in the growth and development of *Sphagnum* mosses, especially in N and C acquisition [22, 24, 65, 91–93]. The results of this study show that HSP had the highest average relative abundance of Rhizobiaceae, which may be related to the lack of N in the ombrotrophic peatland ecosystem; therefore, the *Sphagnum*-associated microorganisms that are linked to N acquisition increase. The genus *Methyloferula* belongs to Beijerinckiaceae, which also contribute to nitrogen fixation [29, 36, 65, 94]. Follow-up work is required where the ^15^N-N_2_ incorporation method is used to verify which bacterial taxa plays a role in N fixation. In addition, cyanobacterial diazotrophs also play certain roles in N fixation in *Sphagnum* moss [25, 29]; however, in this study, Cyanobacteria were not detected in the endophytic bacteriome of the sampled *Sphagnum* mosses. Except for microorganisms with C and N cycling function, chemo-organoheterotrophs were also found in *Sphagnum* mosses. For example, *Granulicella* and *Acidisoma* are known to degrade arabinose and polysaccharides, the main components of cell walls [95, 96]. These genera that are associated with *Sphagnum* mosses and peat soils were isolated from peatland.

Combined with metabolic functional predictions of endophytic bacterial communities, the results showed that both metabolism and genetic information processing pathways were more abundant among the sampled *Sphagnum* mosses (Fig. S5). The results show that the abundances of terpenoid, amino acid, and carbohydrate metabolism genes in the endophytic bacterial community of *Sphagnum* mosses were elevated (Fig. 7b). These pathways are associated with plant growth, photosynthesis, and survival [97]. In a recent study, researchers have identified *Sphagnum* moss metabolites as key factors for microbial structure and characteristics [32]. It remains unclear whether the metabolic pathways with high abundance were related to the “host” release of an array of metabolites to affect the colonization of endophytes or to recruit specific a microbiome [98, 99] and further influence endophytic bacterial metabolic pathways. Therefore, metabolites need to be considered in further experiments to study the relationship with endophytic bacterial in *Sphagnum* mosses.

## Conclusion

In this study, the endophytic microbial community structure and function of different species of *Sphagnum* mosses were examined in two different types of peatlands in China. Significant differences were found in endophytic bacterial communities among different *Sphagnum* mosses, including the same species of *Sphagnum* moss under different types of peatlands. Moreover, methane oxidation rates of methanotrophic populations of *S. palustre* sampled from Hani were higher than those sampled from Taishanmiao. Correspondingly, the average relative abundance of *Methyloferula* (an obligate methanotroph) was higher in SP. Moreover, diazotrophic taxa at the ASV level were also analyzed, and the average relative abundance of Rhizobiaceae was the highest in HSP while other diazotrophs were not present among all mosses. In addition, physiological and biochemical characteristics of mosses driving endophytic bacterial communities were also determined. The remarkable correlation between chlorophyll contents, water-holding capacity, and followed by C contents and endophytic bacterial communities at the relative abundance level of the top 10 phylum, family, and genus. These results enhance the current understanding of dominant endophytic bacteria among *Sphagnum* moss species in Hani and Taishanmiao peatland ecosystems. Many endophytic bacteria in *Sphagnum* mosses are not well known and their functions remain uncertain; therefore, ^15^N-N_2_ fixation experiments are required. Further work should include more species of *Sphagnum* mosses inhabiting different microhabitats, to elucidate the endophytic microbiome, including fungi and archaea. Such knowledge will improve the available understanding of their ecological roles in peatland ecosystems in the face of global climate change.

### Supplementary Information

Below is the link to the electronic supplementary material.

Supplementary file1 (DOCX 3969 KB)

## Data Availability

All 16S rRNA sequences data have uploaded to the Sequence Read Archive of the National Center for Biotechnology Information (NCBI) database and BioProject accession number PRJNA1006557.
