# Supplementary material for: Structure and Functions of Endophytic Bacterial Communities Associated with Sphagnum Mosses and Their Drivers in Two Different Nutrient Types of Peatlands
Source: Microb Ecol. 2024 Feb 26;87(1):47. doi: 10.1007/s00248-024-02355-6 (PMC10896819; doi:10.1007/s00248-024-02355-6)
Supplement: Supplementary file 1 — Supplementary file1 (DOCX 3969 KB) [file 248_2024_2355_MOESM1_ESM.docx]

Supplementary Materials for

**Structure and functions of endophytic bacterial communities associated with *Sphagnum* mosses and their drivers in two different nutrient types of peatlands**

Yue Wang ^a,b,c^, Dan Xue ^a,b,*^, Xuhui Chen ^a,b,c^, Qing Qiu ^a^ , Huai Chen ^a,b,*^

This file includes:

Supplementary Figures 1-5

Supplementary Tables 1-11

**Supplementary figures**


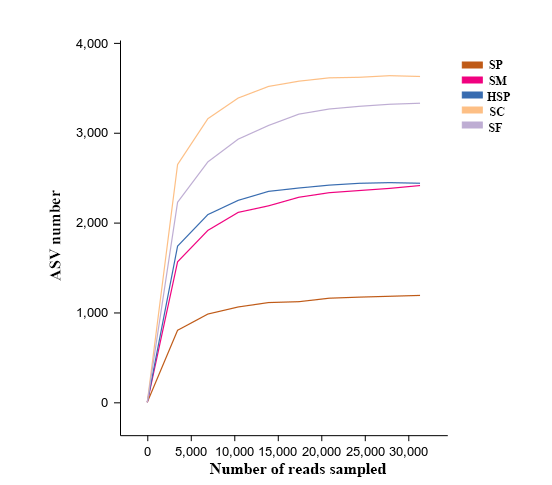


Fig. S1 Rarefaction curves of five *Sphagnum* mosses samples ASV numbers.


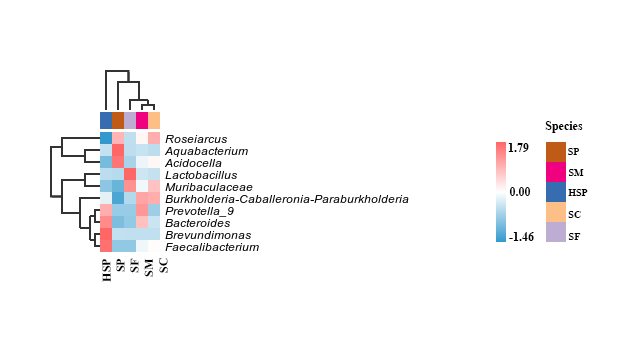


Fig. S2. The heatmap of average relative abundance of the top 10 genera of endophytic bacterium in *Sphagnum* moss.


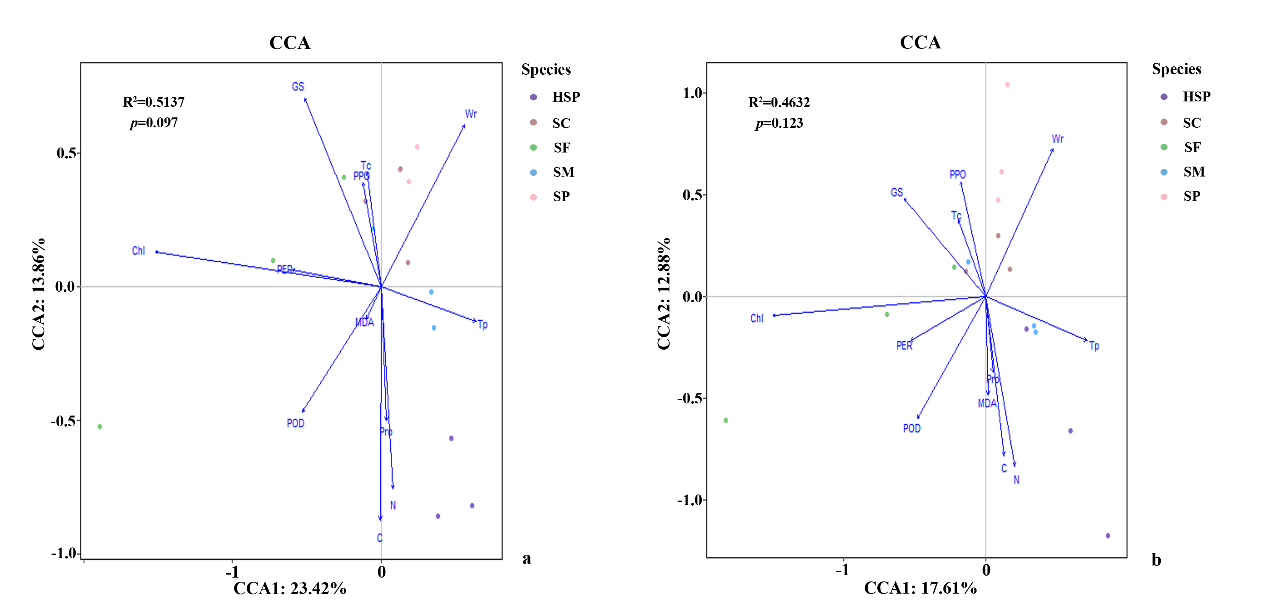


Fig. S3 Canonical correspondence analysis of the average relative abundance of top 10 family (a) and genus (b) endophytic bacterial communities *in Sphagnum* moss in association with *Sphagnum* moss phytochemical parameters. C-total carbon contents; N- total nitrogen contents; Chl-chlorophyll contents; Tp- total phenol contents; Tc- total carbohydrates; Pro-proline; MDA-malondialdehyde; Wr-water retention; PPO-phenol oxidase (PO O_2_); PER-phenol oxidase (PO H_2_O_2_); POD-peroxidase; GS-Glutamine synthetase.


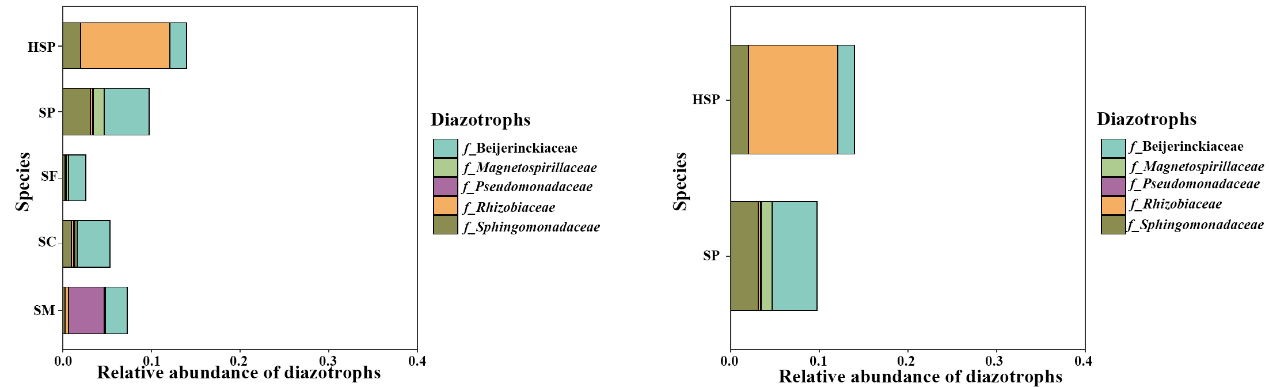


Fig. S4 The relative abundances of putative diazotrophic bacteriome in five species *Sphagnum* moss (a); The relative abundances of putative diazotrophic bacteriome in *Sphagnum.palustre* in Hani (SP) and *Sphagnum.palustre* in Taishanmiao (HSP), respectively (b).


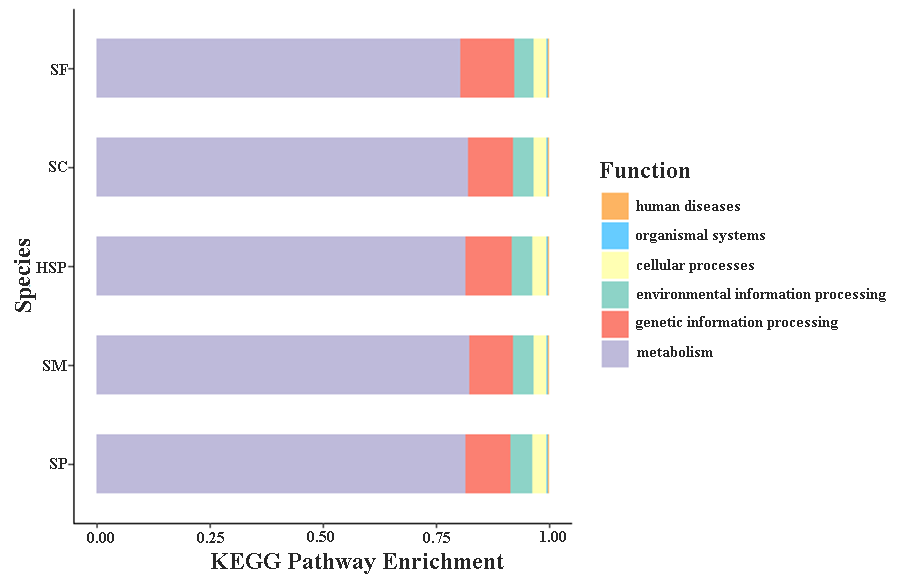


Fig. S5 Relative abundance of the KEGG level I pathways of endophytic bacterium based on PICRUSt2.

**Supplementary Tables**

Table S1 Physiochemical characteristics of peat soil at Hani and Taishanmiao

| Site | TC (%) | TN (%) | C/N | TP (mg g^-1^) | DOC (mg g^-1^) | WC (%) | pH |
| --- | --- | --- | --- | --- | --- | --- | --- |
| Taishanmiao | 30.90±4.21a | 1.65±0.20a | 18.69±0.26a | 1.16±0.12a | 3.04±0.28a | 937.35±110.83a | 5.09±0.14a |
| Hani | 28.71±0.77a | 1.67±0.04a | 17.18±0.80a | 1.02±0.07a | 1.84±0.18b | 643.32±7.43a | 5.71±0.22a |

Values are mean ± SE (n = 3). Values with the lowercase letters indicate significant differences at *p* < 0.05. TC, TN, TP, DOC and WC represent total carbon content, total nitrogen content, total phosphorus content, dissolved organic carbon and peat water content. The C/N values were calculated by the mass ratio of TC/TN.

Table S2 Alpha diversity indices of different *Sphagnum* moss endophytes at Hani and Taishanmiao

| Species | Chao1 | Shannon’s diversity | Pielou’s evenness |
| --- | --- | --- | --- |
| SP | 1201.52±21.82c | 4.97±0.23c | 0.71±0.03b |
| SM | 2418.95±134.84b | 5.90±0.14b | 0.77±0.01ab |
| HSP | 2434.86±237.25b | 5.88±0.18b | 0.76±0.03ab |
| SC | 3625.35±44.64a | 6.84±0.09a | 0.84±0.01a |
| SF | 3315.59±286.53a | 6.17±0.27ab | 0.77±0.03ab |

Values are mean ± SE (n = 3). Values with the lowercase letters indicate significant differences at *p* < 0.05.

Table S3 Average relative abundance of endophytic bacterial communities at the phylum, family and genus level in different *Sphagnum* moss species.

|  | SP | SM | HSP | SC | SF |
| --- | --- | --- | --- | --- | --- |
| *Phylum* |  |  |  |  |  |
| *p_Proteobacteria* | 0.8600±0.0534 | 0.6804±0.0329 | 0.6567±0.0377 | 0.7374±0.0773 | 0.4829±0.1639 |
| *p_Firmicutes* | 0.0040±0.0030 | 0.0665±0.0082 | 0.0997±0.0658 | 0.0690±0.0333 | 0.3467±0.1623 |
| *p_Bacteroidetes* | 0.0043±0.0014 | 0.1741±0.0467 | 0.1646±0.0529 | 0.0796±0.0389 | 0.0836±0.0166 |
| *p_Actinobacteria* | 0.0288±0.0031 | 0.0247±0.0048 | 0.0367±0.0023 | 0.0284±0.0076 | 0.0319±0.0079 |
| *p_Acidobacteria* | 0.0320±0.0107 | 0.0237±0.0043 | 0.0040±0.0002 | 0.0258±0.0036 | 0.0190±0.0069 |
| *p_Deinococcus* | 0.0601±0.0418 | 0.0005±0.0004 | 0.0001±0.0000 | 0.0001±0.0001 | 0.0000±0.0000 |
| *p_Chlamydiae* | 0.0025±0.0008b | 0.0037±0.0011b | 0.0124±0.0008a | 0.0076±0.0035ab | 0.0045±0.0010ab |
| *p_Dependentiae* | 0.0007±0.0005 | 0.0032±0.0015 | 0.0099±0.0078 | 0.0111±0.0023 | 0.0022±0.0010 |
| *p_Armatimonadetes* | 0.0011±0.0006ab | 0.0078±0.0031a | 0.0001±0.0001b | 0.0019±0.0003ab | 0.0020±0.0009ab |
| *p_Verrucomicrobia* | 0.0039±0.0026 | 0.0031±0.0021 | 0.0003±0.0000 | 0.0038±0.0018 | 0.0005±0.0002 |
| *Family* |  |  |  |  |  |
| *f*_*Acetobacteraceae* | 0.2646±0.033a | 0.1725±0.0063ab | 0.0120±0.0009b | 0.2331±0.0340a | 0.2323±0.0865a |
| *f*_*Burkholderiaceae* | 0.2719±0.1106 | 0.2248±0.0284 | 0.1290±0.0176 | 0.1906±0.0218 | 0.0808±0.0264 |
| *f*_*Caulobacteraceae* | 0.0859±0.0184 | 0.0786±0.0157 | 0.2037±0.0855 | 0.0630±0.0176 | 0.0204±0.0053 |
| *f*_*Lactobacillaceae* | 0.0005±0.0003 | 0.0187±0.0107 | 0.0048±0.0030 | 0.0114±0.0085 | 0.2667±0.01287 |
| *f*_*Prevotellaceae* | 0.0008±0.0005 | 0.1068±0.0514 | 0.0982±0.0468 | 0.0091±0.0044 | 0.0086±0.0003 |
| *f*_*Beijerinckiaceae* | 0.0510±0.0062a | 0.0246±0.0025ab | 0.0187±0.0036b | 0.0370±0.0113ab | 0.0187±0.0057b |
| *f*_*Ruminococcaceae* | 0.0012±0.0010 | 0.0206±0.0082 | 0.0671±0.0492 | 0.0304±0.0213 | 0.0184±0.0075 |
| *f*_*Muribaculaceae* | 0.0012±0.0007 | 0.0218±0.0184 | 0.0067±0.0031 | 0.0392±0.0319 | 0.0515±0.0153 |
| *f*_*Rhizobiaceae* | 0.0024±0.0002b | 0.0034±0.0008b | 0.1005±0.0329a | 0.0020±0.0006b | 0.0004±0.0002b |
| *f*_*Bacteroidaceae* | 0.0008±0.0003b | 0.0322±0.0148ab | 0.0437±0.0079a | 0.0112±0.0110ab | 0.0037±0.0005ab |
| *Genus* |  |  |  |  |  |
| *g_Burkholderia-Caballeronia-Paraburkholderia* | 0.0121±0.0032c | 0.1554±0.0266a | 0.0792±0.0220abc | 0.1501±0.0197ab | 0.0577±0.0198bc |
| *g_Lactobacillus* | 0.0005±0.0003 | 0.0187±0.0107 | 0.0048±0.0030 | 0.0114±0.0085 | 0.2667±0.1287 |
| *g_Prevotella_9* | 0.0003±0.0002 | 0.0987±0.0450 | 0.0855±0.0427 | 0.0020±0.0018 | 0.0002±0.0001 |
| *g_Brevundimonas* | 0.0007±0.0001 | 0.0001±0.0001 | 0.1815±0.0883 | 0.0004±0.0000 | 0.0001±0.0001 |
| *g_Aquabacterium* | 0.1196±0.0661 | 0.0054±0.0023 | 0.0042±0.0006 | 0.0015±0.0001 | 0.0022±0.0008 |
| *g_Muribaculaceae* | 0.0012±0.0007 | 0.0218±0.0184 | 0.0066±0.0031 | 0.0392±0.0319 | 0.0513±0.0152 |
| *g_Acidocella* | 0.0594±0.0076a | 0.0194±0.0016bc | 0.0009±0.0003c | 0.0251±0.0064b | 0.0085±0.0040bc |
| *g_Bacteroides* | 0.0008±0.0003b | 0.0322±0.018ab | 0.0437±0.0079a | 0.0112±0.0110ab | 0.0037±0.0005ab |
| *g_Faecalibacterium* | 0.0001±0.0000 | 0.0143±0.0075 | 0.0514±0.0365 | 0.0173±0.0172 | 0.0000±0.0000 |
| *g_Roseiarcus* | 0.0254±0.0029a | 0.0171±0.0019ab | 0.0002±0.0003c | 0.0263±0.0140a | 0.0111±0.0027ab |
| *g_Ralstonia* | 0.00006±0.00005c | 0.0338±0.0028a | 0.0101±0.0039bc | 0.0175±±0.0030b | 0.0088±0.0027bc |
| *g_Granulicella* | 0.0224±0.0083 | 0.0151±0.0028 | 0.0005±0.0003 | 0.0141±0.0045 | 0.0111±0.0038 |
| *g_Deinococcus* | 0.0601±0.0418 | 0.0004±0.0003 | 0.00007±0.00004 | 0.0001±0.00006 | 0.00003±0.00002 |
| *g_Acidisoma* | 0.0182±0.0011a | 0.0171±0.0042a | 0.0012±0.0006b | 0.01360±0.0010a | 0.0096±0.0030ab |
| *g_Allorhizobium-Neorhizobium-Pararhizobium-Rhizobium* | 0.0023±0.0002b | 0.0019±0.0009b | 0.0515±0.0199a | 0.0008±0.0001b | 0.0002±0.0001b |

Values are mean ± SE (n = 3).

Values with the lowercase letters indicate significant differences at *p* < 0.05.

Table S4. Spearman correlations test between the average relative abundance of the top 10 phylum endophytic bacterial of *Sphagnum* moss and physiological and biochemical properties.

|  | C % | N % | Chlorophyll | Total phenols | Total carbohydrate | Proline | MDA | Water hold capacity | GS | PPO | PER | POD |
| --- | --- | --- | --- | --- | --- | --- | --- | --- | --- | --- | --- | --- |
| Proteobacteria | -0.400 | -0.246 | -0.043 | 0.082 | -0.075 | -0.407 | -0.198 | **0.539*** | 0.011 | 0.300 | -0.154 | -0.568 |
| Firmicutes | 0.211 | 0.150 | 0.396 | -0.332 | 0.229 | 0.321 | 0.168 | **-0.520*** | 0.154 | -0.321 | 0.272 | 0.504 |
| Bacteroidetes | -0.021 | 0.204 | -0.286 | 0.114 | 0.107 | 0.186 | 0.377 | -0.089 | -0.175 | -0.439 | -0.038 | 0.532 |
| Actinobacteria | 0.357 | 0.329 | 0.021 | 0.025 | -0.296 | 0.382 | 0.179 | -0.393 | -0.200 | -0.396 | 0.324 | -0.021 |
| Acidobacteria | **-0.604*** | -0.504 | 0.332 | -0.118 | 0.311 | -0.307 | -0.195 | 0.404 | 0.454 | -0.043 | 0.184 | -0.289 |
| Deinococcus | -0.262 | -0.262 | -0.196 | 0.135 | -0.160 | -0.240 | -0.249 | **0.640*** | 0.055 | 0.185 | -0.035 | -0.327 |
| Chlamydiae | 0.371 | **0.657**** | -0.332 | 0.100 | -0.007 | 0.321 | 0.279 | -0.496 | -0.082 | 0.086 | 0.002 | -0.118 |
| Dependentiae | -0.136 | 0.489 | 0.061 | -0.004 | 0.214 | 0.261 | **0.581*** | -0.093 | -0.089 | -0.232 | -0.034 | -0.232 |
| Armatimonadetes | **-0.782**** | **-0.611*** | 0.289 | -0.279 | 0.493 | -0.318 | -0.134 | 0.214 | **0.636*** | -0.393 | 0.290 | 0.321 |
| Verrucomicrobia | **-0.629*** | -0.429 | 0.139 | 0.075 | 0.261 | -0.286 | -0.063 | 0.407 | 0.082 | -0.204 | 0.134 | -0.168 |

Significant correlations are indicated in bold; * *p* < 0.05, ** *p* < 0.01.

Table S5. Spearman correlations test between the average relative abundance of top 10 family endophytic bacterial of *Sphagnum* moss and physiological and biochemical properties.

|  | C% | N% | Chlorophyll | Total phenols | Total carbohydrate | Proline | MDA | Water hold capacity | GS | PPO | PER | POD |
| --- | --- | --- | --- | --- | --- | --- | --- | --- | --- | --- | --- | --- |
| Acetobacteraceae | -0.329 | -0.332 | **0.532*** | 0.139 | 0.129 | -0.425 | 0.036 | 0.271 | 0.479 | 0.186 | 0.234 | -0.396 |
| Burkholderiaceae | **-0.607*** | -0.468 | -0.254 | -0.032 | 0.457 | -0.139 | -0.475 | **0.564*** | 0.075 | -0.110 | -0.440 | -0.243 |
| Caulobacteraceae | 0.000 | 0.136 | **-0.739**** | 0.382 | -0.343 | 0.043 | 0.009 | 0.300 | **-0.561*** | -0.125 | -0.247 | -0.207 |
| Lactobacillaceae | 0.011 | -0.061 | 0.496 | -0.264 | 0.339 | 0.018 | 0.179 | -0.489 | 0.375 | -0.246 | 0.256 | 0.511 |
| Prevotellaceae | 0.143 | 0.343 | -0.425 | 0.307 | 0.036 | 0.175 | 0.347 | -0.196 | -0.254 | -0.389 | -0.075 | 0.471 |
| Beijerinckiaceae | -0.386 | -0.336 | 0.021 | 0.050 | 0.114 | -0.350 | -0.265 | 0.364 | 0.204 | 0.346 | -0.111 | -0.568 |
| Ruminococcaceae | 0.221 | 0.314 | -0.125 | -0.154 | 0.236 | 0.457 | 0.148 | -0.243 | -0.161 | -0.368 | -0.072 | 0.482 |
| Muribaculaceae | 0.000 | 0.046 | **0.525*** | -0.168 | 0.350 | -0.014 | 0.347 | -0.300 | 0.093 | -0.143 | 0.018 | 0.321 |
| Rhizobiaceae | 0.121 | 0.275 | **-0.786**** | 0.321 | -0.232 | 0.150 | -0.013 | 0.089 | **-0.793**** | -0.214 | -0.424 | -0.018 |
| Bacteroidaceae | 0.204 | 0.161 | **-0.521*** | -0.068 | -0.268 | 0.246 | -0.023 | -0.282 | -0.114 | -0.482 | 0.259 | 0.729 |

Significant correlations are indicated in bold; * *p* < 0.05, ** *p* < 0.01.

Table S6. Spearman correlations test between the average relative abundance of the top 10 genus endophytic bacterial of *sphagnum* moss and physiological and biochemical properties.

|  | C% | N% | Chlorophyll | Total phenols | Total carbohydrate | Proline | MDA | Water hold capacity | GS | PPO | PER | POD |
| --- | --- | --- | --- | --- | --- | --- | --- | --- | --- | --- | --- | --- |
| Burkholderia-Caballeronia-Paraburkholderia | **-0.514*** | -0.036 | 0.018 | -0.161 | **0.764**** | 0.106 | 0.055 | 0.111 | 0.211 | -0.45 | -0.197 | 0.189 |
| Lactobacillus | 0.011 | -0.061 | **0.532*** | -0.264 | 0.339 | -0.02 | 0.179 | -0.489 | 0.375 | -0.246 | 0.256 | 0.511 |
| Prevotella_9 | 0.086 | 0.229 | **-0.634*** | 0.244 | -0.168 | 0.09 | 0.029 | 0.104 | -0.168 | -0.226 | 0.007 | 0.301 |
| Brevundimonas | 0.486 | **0.546*** | **-0.65**** | **0.525*** | -0.454 | 0.267 | 0.143 | -0.011 | **-0.739**** | 0.232 | -0.399 | **-0.561*** |
| Aquabacterium | 0.096 | -0.307 | **-0.536*** | 0.279 | **-0.550*** | -0.113 | -0.495 | 0.075 | -0.229 | 0.039 | 0.109 | 0.089 |
| Muribaculaceae | 0 | 0.046 | **0.557*** | -0.168 | 0.35 | -0.043 | 0.347 | -0.3 | 0.093 | -0.143 | 0.018 | 0.321 |
| Acidocella | **-0.625*** | **-0.571*** | 0.182 | -0.121 | 0.186 | -0.304 | -0.366 | **0.750**** | 0.271 | 0.096 | 0.025 | -0.404 |
| Bacteroides | 0.204 | 0.161 | -0.504 | -0.068 | -0.268 | 0.252 | -0.023 | -0.282 | -0.114 | -0.482 | 0.259 | **0.729**** |
| Faecalibacterium | -0.05 | 0.208 | **-0.645**** | 0.057 | 0.072 | 0.088 | -0.097 | 0.208 | -0.412 | -0.215 | -0.366 | 0.215 |
| Roseiarcus | **-0.579*** | -0.475 | 0.307 | 0.05 | 0.321 | -0.487 | -0.157 | 0.504 | 0.332 | 0.336 | -0.102 | **-0.518*** |
| Allorhizobium-Neorhizobium-Pararhizobium-Rhizobium | 0.246 | 0.254 | **-0.771**** | 0.346 | -0.436 | 0.043 | -0.097 | 0.054 | **-0.800**** | 0.029 | -0.411 | -0.179 |

Significant correlations are indicated in bold; * *p* < 0.05, ** *p* < 0.01.

Table S7. The average relative abundances of methanotrophic prokaryotic taxa in *Sphagnum* moss endophytic bacteriome

|  |  | SP | | HSP | | SM | | SC | | SF | | Kruskal Wallis test |
| --- | --- | --- | --- | --- | --- | --- | --- | --- | --- | --- | --- | --- |
|  | | mean | se | mean | se | mean | se | mean | se | mean | se | *p* value |
| Methanotrophs | |  |  |  |  |  |  |  |  |  |  |  |
| *f*_*Beijerinckiaceae* | | 0.051 | 0.0061 | 0.0187 | 0.0035 | 0.0246 | 0.0025 | 0.037 | 0.0112 | 0.0187 | 0.0057 | 0.081 |
| *g*_*Methylobacterium* | | 0.004 | 0.0006 | 0.0124 | 0.0036 | 0.002 | 0.0006 | 0.002 | 0.0008 | 0.001 | 0.0004 | 0.032 |
| *g*_*Methyloferula* | | 0.0058 | 0.0029 | 0.00004 | 0.00002 | 0.0018 | 0.0005 | 0.0008 | 0.0002 | 0.0026 | 0.0008 | 0.038 |

Table S8. The average relative abundances of nitrogen fixation prokaryotic taxa in *Sphagnum* moss endophytic bacteriome

|  |  | SP | | HSP | | SM | | SC | | SF | | Kruskal Wallis test |
| --- | --- | --- | --- | --- | --- | --- | --- | --- | --- | --- | --- | --- |
|  | | mean | se | mean | se | mean | se | mean | se | mean | se | *p* value |
| Diazotrophs | |  |  |  |  |  |  |  |  |  |  |  |
| *f*_*Beijerinckiaceae* | | 0.051 | 0.0061 | 0.0187 | 0.0035 | 0.0246 | 0.0025 | 0.037 | 0.0112 | 0.0187 | 0.0057 | 0.081 |
| *f*_*Magnetospirillaceae* | | 0.0125 | 0.0052 | 0.0004 | 0.0002 | 0.0013 | 0.0006 | 0.0029 | 0.0019 | 0.0025 | 0.0013 | 0.073 |
| *f*_*Sphingomonadaceae* | | 0.0311 | 0.0096 | 0.0202 | 0.0033 | 0.0031 | 0.0008 | 0.0099 | 0.0057 | 0.0027 | 0.0009 | 0.045 |
| *f*_*Rhizobiaceae* | | 0.0024 | 0.0002 | 0.1005 | 0.0329 | 0.0034 | 0.0008 | 0.002 | 0.0006 | 0.0003 | 0.0002 | 0.213 |
| *f*_*Pseudomonadaceae* | | 0.0008 | 0.0005 | 0.0001 | 0.00003 | 0.0406 | 0.0381 | 0.0009 | 0.0004 | 0.003 | 0.0001 | 0.024 |

Table S9 Comparison of methane oxidation activity (^13^C-CH_4_ incorporation rate) in *Sphagnum* moss were sampled in Hani (SP) and Taishanmiao (HSP).

| Species | Light + Dark (48 h) | Dark (48 h) |
| --- | --- | --- |
| HSP | 2.28 ± 0.83 | 5.91 ± 0.24 |
| SP | 3.07 ± 0.26 | 6.25 ± 0.99 |

Values are mean ± SE (n = 3).

Light/dark treatment represents moss samples were in 100 ml transparent sterile bottle and kept in artificial climate chamber for 16h at day and 8h at night at 24℃. Dark treatment represent moss samples were in 100 ml brown sterile bottle and kept in artificial climate chamber without light for 48h at 24℃.

Table S10. Spearman correlations test showing the relative abundance of the top 10 family endophytic bacterium of *Sphagnum* moss association with predicted function by FAPROTAX

|  | Acetobacteraceae | Burkholderiaceae | Caulobacteraceae | Lactobacillaceae | Prevotellaceae | Beijerinckiaceae | Ruminococcaceae | Muribaculaceae | Rhizobiaceae | Bacteroidaceae |
| --- | --- | --- | --- | --- | --- | --- | --- | --- | --- | --- |
| Ureolysis | **-0.685^**^** | 0.214 | 0.499 | -0.064 | **0.702**** | -0.345 | 0.484 | -0.041 | **0.794**** | 0.606 |
| Methylotrophy | -0.218 | -0.079 | **0.546*** | **-0.543*** | -0.089 | 0.150 | -0.218 | **-0.564*** | 0.489 | 0.121 |
| Methanol_oxidation | -0.304 | 0.079 | **0.757**** | **-0.55*** | -0.021 | 0.150 | -0.154 | **-0.55*** | **0.693**** | 0.064 |
| Methanotrophy | **0.547*** | 0.202 | -0.374 | -0.077 | **-0.577*** | -0.429 | -0.420 | -0.166 | -0.433 | -0.334 |
| Nitrogen_fixation | -0.370 | 0.164 | **0.677**** | **-0.672**** | 0.063 | 0.080 | 0.048 | -0.466 | **0.786**** | 0.172 |
| Nitrification | -0.090 | 0.086 | 0.075 | 0.136 | **0.609*** | -0.244 | 0.326 | 0.197 | 0.204 | 0.280 |
| Nitrate_reduction | **-0.668**** | -0.157 | 0.057 | 0.232 | **0.721**** | **-0.625*** | **0.7**** | 0.254 | 0.511 | **0.739**** |
| Aerobic_ammonia_oxidation | -0.129 | 0.014 | 0.204 | 0.131 | 0.504 | -0.274 | 0.326 | 0.257 | 0.423 | 0.201 |
| Aerobic_nitrite_oxidation | -0.213 | 0.032 | 0.014 | 0.111 | **0.748**** | -0.280 | 0.456 | 0.222 | 0.163 | 0.380 |
| Nitrite_respiration | -0.207 | 0.263 | 0.122 | 0.066 | 0.353 | -0.174 | **0.537*** | 0.405 | **0.55*** | 0.079 |
| Nitrate_respiration | -0.313 | 0.309 | 0.040 | 0.164 | **0.593*** | -0.255 | **0.589*** | 0.411 | **0.524*** | 0.360 |
| Nitrogen_respiration | -0.316 | 0.316 | 0.069 | 0.127 | **0.582*** | -0.236 | **0.586*** | 0.389 | **0.542*** | 0.342 |
| Photoautotrophy | -0.083 | 0.361 | -0.050 | 0.000 | 0.268 | 0.190 | 0.159 | 0.393 | 0.188 | -0.006 |
| Phototrophy | 0.153 | 0.446 | -0.047 | -0.118 | 0.058 | 0.430 | -0.149 | 0.242 | 0.058 | -0.100 |
| Xylanolysis | **-0.746**** | -0.061 | 0.114 | 0.182 | **0.832**** | **-0.718**** | **0.786**** | 0.186 | 0.443 | **0.921**** |
| Fermentation | -0.454 | -0.379 | **-0.514*** | **0.721**** | 0.489 | **-0.775**** | **0.85**** | **0.607*** | -0.146 | **0.532*** |
| Aerobic_chemoheterotrophy | 0.029 | 0.386 | **0.561*** | **-0.689**** | -0.361 | 0.371 | -0.339 | -0.461 | **0.554*** | -0.079 |
| Hydrocarbon_degradation | 0.361 | 0.391 | -0.245 | -0.059 | -0.191 | 0.097 | -0.009 | 0.066 | -0.132 | -0.066 |
| Aromatic_compound_degradation | 0.406 | 0.322 | 0.194 | **-0.694**** | **-0.646**** | **0.597*** | **-0.646**** | **-0.564*** | -0.054 | -0.470 |

Significant correlations are indicated in bold; * *p* < 0.05, ** *p* < 0.01.

Table S11. Metabolism and genetic pathways (KEGG Ⅱ) of endophytic bacterium by PICRUSt2.

|  | SP | SM | HSP | SC | SF |
| --- | --- | --- | --- | --- | --- |
| **Level Ⅰ** |  |  |  |  |  |
| Human diseases | 199.41 | 171.53 | 183.8 | 178.93 | 182.68 |
| Organismal systems | 118.63 | 156.41 | 135.86 | 139.86 | 111.14 |
| Cellular processes | 1253.86 | 1098.15 | 1229.17 | 1169.3 | 1076.31 |
| Environmental informaition processing | 1935.88 | 1871.35 | 1878.68 | 1857.57 | 1660.07 |
| Genetic infromaition processing | 4162.81b | 3863.33b | 4115.22 b | 4119.36 b | 4712.98 a |
| Metabolism | 31171.01 | 31365.92 | 30661.21 | 32032.87 | 29571.94 |
| **Level Ⅱ in metabolism** |  |  |  |  |  |
| Carbohydrate metabolism | 5527.53 | 5676.97 | 5476.04 | 5720.75 | 5587.42 |
| Energy metabolism | 2102.54 | 1950.94 | 2017.74 | 2037.78 | 1954.3 |
| Lipid metabolism | 2287.46 | 2295.64 | 2197.07 | 2333.67 | 2694.79 |
| Nucleotide metabolism | 584.84 | 556.2 | 589.5 | 581.99 | 704.92 |
| Amino acid metabolism | 5398.15 | 5251.46 | 5267.18 | 5382.49 | 4716.79 |
| Metabolism of other amino acids | 2189.8 | 2045.76 | 1982.55 | 2096.86 | 1873.71 |
| Glycan biosynthesis and metabolism | 1083.73 | 1189.49 | 1168.87 | 1142.75 | 1117.58 |
| Metabolism of cofactors and vitamins | 4681.79 | 4365.91 | 4449.19 | 4575.66 | 4081.77 |
| Metabolism of terpenoids and polyketides | 3175.29 ab | 3516.22 ab | 3407.95 ab | 3648.31 a | 2993.68 b |
| Biosynthesis of other secondary metabolites | 838.2 | 894.03 | 930.2 | 909.72 | 805.55 |
| Xenobiotics biodegradation and metabolism | 2911.53 | 3278.45 | 2803.94 | 3244.89 | 2776.62 |
| Chemical structure transformation maps | 407.7 | 360.7 | 387.52 | 371.13 | 274.12 |
| **Level Ⅱ in genetic information processing** |  |  |  |  |  |
| Transcription | 274.76 | 281.89 | 310.51 | 291.73 | 281.95 |
| Translation | 940.79 | 859.35 | 906.65 | 934.29 | 1143.95 |
| Folding, sorting and degradation | 1170.29 | 1025.63 | 1052.74 | 1115.38 | 1175.11 |
| Replication and repair | 1776.97 | 1696.46 | 1845.32 | 1777.96 | 2111.96 |
